# All-cause and cause-specific mortality trends among people with and without HIV in the Siaya health and demographic surveillance system, Kenya, 2011–2018

**DOI:** 10.1080/16549716.2026.2640299

**Published:** 2026-03-05

**Authors:** Julie Ambia, Adam Trickey, Suzanne M. Ingle, Kathryn Risher, Fredrick Odongo, Georges Reniers, Daniel Kwaro

**Affiliations:** a Population Health Sciences, University of Bristol, Bristol, UK; b Department of Public Health Sciences, Penn State University College of Medicine, Hershey, PA, USA; c Kenya Medical Research Institute – Center for Global Health Research, Kisumu, Kenya; d Department of Population Health, London School of Hygiene and Tropical Medicine, Bristol, UK; e MRC/Wits Rural Public Health and Health Transitions Research Unit (Agincourt), School of Public Health, Faculty of Health Sciences, University of the Witwatersrand, Johannesburg, South Africa

**Keywords:** Cause of death, HDSS, mortality hazard, Joinpoint regression, verbal autopsy

## Abstract

**Background:**

All-cause mortality among people with HIV (PWH) in sub-Saharan Africa declined after antiretroviral therapy’s introduction, but data in rural settings on evolving causes of death as this population age remain limited.

**Objectives:**

To compare all-cause and cause-specific mortality trends among PWH and people without HIV (PWOH) in western Kenya using a prospective cohort study.

**Methods:**

Data from the Siaya Health and Demographic Surveillance System were used to estimate mortality rates from 2011 to 2018 among persons aged 15–64 years, with the study population (PWH/PWOH) determined through HIV testing. InterVA-4 was used to ascertain the cause of death.

**Results:**

45,581 individuals with an HIV test result contributed 209,078 person-years (py) of follow-up. The HIV prevalence was 14.5%. Median age among PWH increased from 37 to 42 years from 2011 to 2018. For PWOH, this was between 29 and 31 years. 1386 individuals died, 48.8% were PWH. HIV/AIDS/tuberculosis (319 deaths; 58.2%) was the leading mortality cause for PWH and non-communicable diseases (NCDs) (235; 40.9%) for PWOH. From 2011 to 2017, HIV/AIDS/tuberculosis mortality rates declined among PWH from 19.0 to 7.0 deaths/1,000py, and mortality due to NCDs increased from 3.7 in 2014 to 5.1/1,000py in 2017. For PWOH, cause-specific mortality trends were stable over time.

**Conclusion:**

Among PWH, HIV/AIDS/tuberculosis mortality decreased from 2011 to 2017, while mortality rates due to NCDs rose over time as the population aged. Among PWOH, NCDs were the leading cause of death. Managing HIV and the increasing burden of NCDs in this community requires education on prevention, active screening, and delivery of treatment and palliative care services.

## Paper Context

- **Main findings:** This analysis shows the progress made in reducing AIDS-related mortality among people living with HIV in western Kenya and an increase in non-communicable disease mortality rates in an ageing HIV population.
- **Added knowledge:** Considering the improved survival and increasing age of people living with HIV, age-related non-communicable disease mortality could continue rising over time among individuals with HIV.
- **Global health impact for policy and action:** Implementing educational campaigns focused on modifying risk factors associated with non-communicable diseases, and early detection is crucial for improving health outcomes among people living with HIV in sub-Saharan Africa.

## Background

Kenya’s first national household survey in 2003 estimated that the HIV prevalence in the Nyanza province, which included Siaya County, was 15%, compared to 7% nationally [1], with follow-up surveys in 2007 and 2012 showing similar results [2]. In 2018, among 47 Kenyan counties, Siaya had the third highest HIV prevalence in a nationwide survey, with 15% of individuals aged 15–64 living with HIV, compared to the 5% national prevalence [3].

Despite a stable HIV prevalence, these two decades were marked by a decline in HIV incidence and HIV/AIDS-related mortality rates in Nyanza and Siaya [4,5]. The life expectancy at birth in Siaya County rose from 47 years in 2006 to 65 years in 2016 [5]. Additionally, between 1990 and 2016, Siaya saw the fastest decline in all-cause and HIV/AIDS-related mortality among Kenya’s 47 counties [5]. Despite mortality declining, in 2019, Siaya County still had the highest all-cause crude mortality rate, 15.5 deaths per 1000 population, compared to national estimates of 10.5 [6].

The decline in HIV/AIDS-related mortality has mainly been attributed to the increased scale-up of antiretroviral therapy (ART) for HIV in the early 2000s [7]. In Siaya County, free ART services were first introduced in a large public hospital in 2004, with subsequent expansion to smaller hospitals and health centers [8]. By December 2006, eight health facilities within the Siaya Health and Demographic Surveillance System (HDSS) catchment area were providing ART services, increasing to 42 in 2016 [7,9]. ART coverage among people with HIV (PWH) increased from 1% in 2005 to 64% in 2008 based on the criterion of <250 CD4 cells/μL for ART eligibility [7].

The Gem region of the Siaya HDSS reported an increase in ART coverage from 32% to 56% between 2011 and 2016 [10]. During this period, Kenya adopted the World Health Organization (WHO) 2010 guidelines, which raised the eligibility threshold for ART to a CD4 count of <350 cells/μL or a WHO clinical stage of III or IV disease [11]. This eligibility threshold was further increased to a CD4 count of <500 cells/μL between 2014 and 2016, following the WHO 2013 recommendations [12]. In 2016, Kenya implemented a ‘universal test and treat’ strategy, offering ART to all individuals living with HIV, regardless of their CD4 count or clinical stage, in accordance with the WHO 2016 guidelines [13].

In the Gem region in Siaya HDSS, HIV incidence in the overall population decreased from 11 to 6 per 1000 person-years between 2011 and 2016, while all-cause mortality rates dropped from 10.0 to 7.0 per 1000 person-years during the same period [10,14]. As the risk of dying from AIDS-related conditions declines among PWH following the wider availability of ART, there is an increasing probability of dying from age-related conditions that are commonly seen among people without HIV (PWOH) [15].

The Siaya HDSS is a well-established population surveillance platform situated in Siaya County, western Kenya. It offers a valuable opportunity to monitor mortality patterns over time. Through continuous follow-up of individuals, regular HIV testing, and the collection of vital statistics, the platform facilitates the analysis of changes in population demographics and causes of death using verbal autopsies. Using data from the Siaya HDSS, the objectives of this study were to 1) assess trends in all-cause and cause-specific mortality from 2011 to 2018 by HIV status, and 2) assess how the rate of dying from different causes has evolved over time while accounting for demographic changes between PWH and PWOH.

## Methods

### Study design and area

This was a prospective cohort study design. From 2001 to the present, twice-yearly or quarterly demographic information on births, deaths, and migrations has been collected from the people residing within the boundaries of Siaya HDSS [16]. Located at latitude −0.09 and longitude 34.77 northeast of Lake Victoria in western Kenya, the HDSS is 700 square kilometres in size and includes 385 villages spread across three regions: Gem, Asembo, and Karemo [16]. The main sources of livelihood in the HDSS are farming, fishing, and small-scale trading [16]. Over time, the HDSS residents’ living standards have been gradually improving [17]. Within Siaya County, the poverty rate, measured as the percentage of people living below the poverty line, fell from 34% in 2015/2016 to 25% in 2022 [18,19].

### Collection of vital statistics

During data collection, a proxy respondent reports any births, deaths, or migrations that occurred in the household since the last HDSS data round to the fieldworker. The collection of vital statistics within the HDSS is still ongoing in 2026. However, in this analysis, we only included data until 31 December 2018, corresponding to the conclusion of another study project that was sponsored by the Analysing Longitudinal Population-based HIV/AIDS data on Africa (ALPHA) network [20].

### Siaya health and demographic surveillance system population

The demographic profile of the population has been previously outlined [16]. In brief, from 2003 to 2008, the birth rate stayed steady at 37 per 1000 residents, while the mortality rate declined from 24 to 19 per 1000 residents [16]. During the same period, life expectancy at birth increased from 38 to 45 years [16].

People who move into the HDSS area are first registered for four months on a temporary basis. Their permanent residency status was then validated during the next HDSS round of data collection [21].

### Ascertaining cause of death

Deaths are reported by village informants who liaise with a field worker to conduct a verbal autopsy within three months of the event [16]. Verbal autopsies were done with the WHO verbal autopsy questionnaire that was adapted by the International Network for the Demographic Evaluation of Populations and Their Health (INDEPTH) [22].

In this analysis, the InterVA-4 probabilistic model was used to assign the cause of death [23]. The InterVA has been used to evaluate mortality trends among PWH in a number of countries, including South Africa [24]. Furthermore, a validation study conducted in sub-Saharan Africa found that the InterVA-4 had a 90% specificity in attributing deaths to HIV/AIDS [23].

The InterVA-4 outputs three potential causes of death per individual with their likelihood scores, designating the one with the highest likelihood as the main cause [23]. The unclassifiable category was allocated when the likelihood value for any cause of death was less than 0.4 for an individual [25].

Causes of death were classified into six categories as follows: HIV/AIDS/tuberculosis, infectious diseases, non-communicable diseases (NCDs), external causes/direct obstetric-related, unclassifiable deaths, and missing cause of death. HIV/AIDS and tuberculosis deaths were merged into one group as the verbal autopsies were limited in their ability to distinguish deaths due to tuberculosis that were not AIDS-related [26]. The HIV testing results from the serosurveys were not used to determine HIV/AIDS deaths. External causes and direct obstetric-related deaths were grouped together due to the small numbers of deaths recorded in these categories. The individuals who lacked verbal autopsy data were classified under the missing cause of death category.

### Description of population residing within the gem region of the Siaya HDSS

Among the three regions (Gem, Asembo, and Karemo) in the HDSS, repeated HIV serosurveys have been conducted in Gem. The following section of this analysis is limited to the Gem region since the main objective was to assess cause-specific mortality trends by HIV status. Approximately 100,000 residents live within the Gem region in Siaya HDSS. Further details of the population description are on Appendix page 1 and Appendix Tables A1 and A2.

### HIV serosurvey

In 2010, 40,000 out of 57,000 people aged ≥13 years and residing in 7000 compounds that were randomly chosen through a multi-stage sampling method participated in the first HIV serosurvey in the Gem region, Siaya HDSS [27]. All individuals who resided in the household the night preceding the HIV serosurvey and consented to participate were deemed eligible [28]. In addition, persons who were not HDSS residents but were present in the sampled compound on the night before the survey and met the inclusion criteria took part in the HIV serosurvey after providing informed consent [28].

A two-step random sampling procedure was used to choose the 7,000 compounds from the 14,500 that were part of the sampling frame [28]. First, one of the 25 community leaders drew a paper with a unique compound registration number from a bucket [28]. Then, the study statisticians picked a random number generated by a computer, repeating this process until 50% of all compounds had been sampled [27]. After 2012, the study statisticians used simple random sampling to include more compounds because the number of people randomly chosen to participate in the survey was declining due to deaths or out-migration [29].

The changes in the population structure through death, out-migration, and reaching 65 years old of the people that participated in the HIV serosurveys are discussed in Appendix page 3 and Appendix Table A3. Additionally, the age, sex, and HIV prevalence of individuals that were included in these two random sampling groups are discussed in Appendix page 3 and presented in Appendix Table A4. The number of individuals who participated in the six HIV serosurveys ranged from 15,475 in 2018 to 39,137 in 2010–2011 (Table 1).

**Table 1.** HIV testing algorithms and the number of individuals participating in HIV serosurveys in the Siaya health and demographic surveillance system.

Year	HIV testing algorithm	First HIV test	Second HIV test	Third (tie breaker)	Number of individuals with HIV test results	Reference for HIV testing algorithm
2010–2011	Parallel testing	Determine (Alere, Orlando, FL, USA)	SD Bioline HIV-1/2 3.0 (Standard Diagnostics, Giheung-gu, Gyonggi-do, South Korea)	Uni-gold (Trinity Biotech, Bray, Wicklow, Ireland)	39,137	[14]
2012	Parallel testing	Determine (Alere, Orlando, FL, USA)	SD Bioline HIV-1/2 3.0 (Standard Diagnostics, Giheung-gu, Gyonggi-do, South Korea)	Uni-gold (Trinity Biotech, Bray, Wicklow, Ireland)	21,233	[14]
2013–2014	Serial testing	KHB Colloidal Gold (KHB Shanghai Kehua Bio-engineering Co,Shanghai, China)	*First Response HIV 1–2.O*™ (Premier Medical Corporation Ltd., Kachigam, India)	Uni-gold (Trinity Biotech, Bray, Wicklow, Ireland)	28,132	[30]
2016	Serial testing	Determine (Alere, Orlando, FL, USA)	*First Response HIV 1–2.O*™ (Premier Medical Corporation Ltd., Kachigam, India)	Uni-gold (Trinity Biotech, Bray, Wicklow, Ireland)	27,578	[14]
2017	Serial testing	*Alere Determine*^*TM*^ *HIV-1/2* (Alere Medical Co. Ltd, Chiba, Japan)	*First Response HIV 1–2.O*™ (Premier Medical Corporation Ltd., Kachigam, India)	DNA polymerase chain reaction (PCR)	17,174	[31]
2018	Serial testing	*Alere Determine*^*TM*^ *HIV-1/2* (Alere Medical Co. Ltd, Chiba, Japan)	*First Response HIV 1–2.O*™ (Premier Medical Corporation Ltd., Kachigam, India)	DNA polymerase chain reaction (PCR)	15,475	[27]

### HIV testing procedure

The HIV testing algorithm used in the Siaya HDSS was based on the national HIV testing guidelines established by Kenya’s Ministry of Health (Table 1). Every individual who was tested for HIV received both pre- and post-test counselings. However, individuals with HIV who had documented HIV test results were not re-tested. Therefore, in this analysis, testing HIV-positive during the serosurvey or having documentation of a positive HIV test from the medical facility was used to define positive HIV status, while testing negative during the serosurvey was used to indicate HIV-negative status.

### Time-varying HIV status

We allowed a person’s HIV status (negative, positive, and unknown) to vary over time based on their HIV test results, with the date of a status change assumed to happen between test dates. For a person who first had a negative HIV test and then tested positive, the probable date of seroconversion was considered as the midpoint between their last negative date and first positive date. For a person who had two negative HIV tests, this person was considered HIV negative in between the two tests regardless of the amount of time that had elapsed. For a person who tested HIV-negative and for whom there was no subsequent HIV testing information, this individual was assumed to be HIV-negative for five years, and thereafter, their HIV status was categorized as unknown, because five years was assumed to be the median time that 5% of the population would have seroconverted [20]. An individual with a positive HIV test maintained a consistent positive HIV status thereafter. The minimum number of HIV tests an individual may have had throughout the follow-up period was one, while the highest was six.

### Additional information regarding datasets used in this analysis

This analysis uses the Siaya HDSS datasets listed below. Only the variables from the datasets utilized in this analysis are reported. The demographic surveillance residency episodes dataset contained information on entry and exit dates, date of birth, sex, and region of residence. The HIV serosurvey dataset included the date of the HIV test, the HIV test results, and the HIV serosurvey name. The verbal autopsy dataset included the main cause of death. The anonymized individual identification numbers were then used to link the residency datasets to the HIV serosurvey dataset and the verbal autopsy dataset.

### Eligibility criteria for analysis inclusion

This analysis used data from individuals aged 15–64 who lived in the Gem region at any time between January 2011 and December 2018 and had at least one HIV test result. These individuals are referred to as the study population. We included verbal autopsy data from 2011 to 2017, since cause of death data were unavailable in 2018. Due to the amount of mortality data available per calendar year, age was categorized as 15–34, 35–49, and 50–64 years.

### Participant follow-up

Follow-up started at the time of a person’s first HIV test. For all individuals who had an HIV test between October and December 2010, follow-up time started at 1 January 2011, and this was done to maximize the amount of HIV status data available. For PWH, follow-up ended at the earliest of the date a person turned 65 years old, death, migration out of the area, or administrative censoring on 31 December 2018. For PWOH, this was the same, but follow-up could also end when an individual’s HIV status changed to unknown or positive.

## Analysis

### Descriptive statistics

To describe participant characteristics, categorical variables including age, gender, HIV status, and number of deaths were presented as counts and percentages, whereas continuous variables such as age were summarized using medians (interquartile ranges).

### Crude mortality rates

Crude all-cause and cause-specific mortality rates, which were expressed as deaths per 1000 person-years at risk, were calculated by dividing the number of deaths from a given cause that occurred during a given calendar year by the total number of person-years of observation contributed by the study population. To compare cause-specific mortality trends over time and between HIV-positive and HIV-negative populations, crude mortality rates were calculated separately for each cause of death and calendar year.

### Joinpoint regression

Age-standardized rates and the annual percentage change (APC) in both all-cause and cause-specific mortality over time by HIV status were estimated using the Joinpoint Regression Program version 5.3.0 [32]. The input data was organized as outlined below. For both all-cause and cause-specific mortality analyses, the denominators used were the total population within each age group. Where no deaths were recorded in any age-group, 0.5 was added to avoid the problem of empty cells (Table A5). Unclassifiable deaths were not included as a category in the Joinpoint regression analyses as there were too few to be analyzed. However, unclassifiable deaths were included in the all-cause mortality model.

Age-specific mortality rates were then computed by dividing the number of deaths in each age group (from all causes or from a specific cause) by the corresponding age-specific population. Next, these rates were applied to a standard population age distribution, 2013 INDEPTH Sub-Saharan Africa standard population, to derive the age-standardized rates [33].

For the Joinpoint program analysis, mortality rates were first log-transformed so that they were normally distributed. The Bayesian Information Criterion (BIC) was then used to assess the number of segments where mortality rates changed over time [34] (Table A6). Joinpoint regression applies the Monte Carlo Permutation method, to segment the age-standardized rates based on differing trends [35]. Age-standardized all-cause and cause-specific mortality rates by HIV status were estimated for every calendar year.

### Survival models

We utilized Cox and competing risk survival models to assess how changing demographics influence all-cause and cause-specific mortality trends. Calendar year was treated as a continuous variable in the regression models to assess temporal trends in all-cause and cause-specific mortality risk. This allowed us to estimate the hazard ratio for each additional year (per one-year increase), facilitating the identification of linear trends across the study period. The calendar year was modeled as continuous and centered on 2015 to decrease multicollinearity because the model included interaction terms [36].

In the univariable survival models, time (calendar year) was the primary exposure variable, whereas cause of death was the outcome. The dependent variable in the multivariable models was cause of death, whilst age, sex, and calendar year were the independent variables.

Cox regression models were used to analyze all-cause mortality hazard ratios for each calendar year. Competing risks regression was used to model subdistribution of mortality hazard ratios for specific causes of death, accounting for other competing causes of death. To estimate the competing risk regression coefficient in the Fine and Gray model, the risk set is constructed to include both individuals who have not experienced any event and those who have undergone a competing event [37]. During follow-up, the number of individuals increases in the risk set, compared to what would be observed in a standard survival framework without competing risks. As the risk set now comprises individuals who have experienced a competing event [37]. This is based on the assumption that they would still be under observation in the absence of a competing event [37].

To conduct the competing risks survival analysis, a specific cause of death was the event of interest, and the competing events were the other five causes of death, including the missing and unclassifiable causes of death categories. For instance, if the primary event was death due to HIV/AIDS/tuberculosis, and the competing risks included death from other causes including infectious diseases, NCDs, external/obstetrics causes, as well as unclassifiable, and those missing a cause of death. Note, a missing cause of death was also included as a cause of death.

To estimate the effect of calendar year on mortality by HIV status, the mortality hazard and subdistribution hazard models were run separately for PWH and PWOH. To assess how differences in mortality among PWH and PWOH change over time, an additional regression model included an interaction term between HIV status and calendar year, adjusting for age and sex.

The proportionality of hazards was assessed using Schoenfeld residuals after fitting the Cox regression model (Table A7). In competing risks regression, the proportional subdistribution hazard assumption was assessed by including an interaction between all covariates and analysis time and assessing the *p*-values of this interaction term. Stata 18.0 was used in this analysis [38].

### Sensitivity analysis

A sensitivity analysis was conducted to compare the mortality hazard ratios when assuming the person time remaining HIV-negative after testing was two years instead of five years, as previously reported [20].

### Ethics and consent

The study received ethical approval from the Scientific Ethics Review Unit of the Kenya Medical Research Institute (Ref No. 1801 and 3589) and the Institutional Review Board of the London School of Hygiene and Tropical Medicine (Ref No. 14,458). Eligible individuals aged 18 years and older were asked to provide written informed consent in Dholuo, English, or Kiswahili. For participants aged 15 to 17 years, parental consent was requested along with the minor’s assent. The study was carried out in accordance with the principles of the Declaration of Helsinki.

## Results

### Participant characteristics

Overall, 45,581 people comprised the study population, of whom 56.1% were women. The person-years of observation contributed by PWH and PWOH were 30,286 and 178,792, respectively (Table 2). The HIV prevalence increased from 13.5% in 2011 to 16.5% in 2018, averaging 14.5% across the years. By sex, from 2011 to 2018, HIV prevalence increased from 15.9% to 19.6% among women and 10.8% to 12.8% among men.

**Table 2.** Summary of the study population by HIV status: total number, person-years, and median age in the Siaya health and demographic surveillance system.

Year	Total number of people	Total number of people with HIV	HIV prevalence	Person-years of observation among people with HIV	Median age (IQR) of persons with HIV	Median age (IQR) at death of persons with HIV	Person-years of observation among people without HIV	Median age (IQR) of persons without HIV	Median age (IQR) at death of persons without HIV
2011	28,763	3,895	13.5	2,581	37 (29–47)	43 (32–54)	16,438	29 (20–46)	52 (33–61)
2012	28,618	3,906	13.6	3,470	37 (30–47)	38 (32–49)	21,984	28 (19–45)	49 (32–60)
2013	31,943	4,336	13.6	3,855	38 (30–48)	42 (33–50)	24,242	27 (19–44)	53 (35–61)
2014	31,028	4,152	13.4	4,010	39 (31–48)	41 (33–50)	25,250	28 (19–44)	46 (29–60)
2015	29,425	3,917	13.3	3,806	40 (32–49)	44 (38–51)	24,327	29 (20–45)	50 (30–59)
2016	31,751	4,633	14.4	4,046	41 (33–50)	40 (32–50)	23,621	30 (20–45)	47 (29–56)
2017	28,260	4,478	15.5	4,301	41 (34–50)	45 (34–55)	22,431	31 (21–46)	52 (34–60)
2018	27,881	4,690	16.5	4,217	42 (35–51)	45 (37–50)	20,499	31 (20–46)	43 (29–60)
			14.5*	**30,286†**	39 (32–49)*	42 (34–52)*	**178,792†**	29 (20–45)*	50 (32–60)*

IQR: Interquartile range; *Average across years; **†**Total.

### Changes in study population structure by age and year

PWH was older than PWOH, with a median age of 39 years (Interquartile range (IQR): 32–49) among PWH and 29 years (IQR: 20–45) among PWOH. The median age among PWH increased from 37 years (29–47) in 2011 to 42 years (35–51) in 2018. Among PWOH, the median age increased from 29 years (IQR: 20–46) in 2011 to 31 years (IQR: 20–46) in 2018.

**Figure 1.**
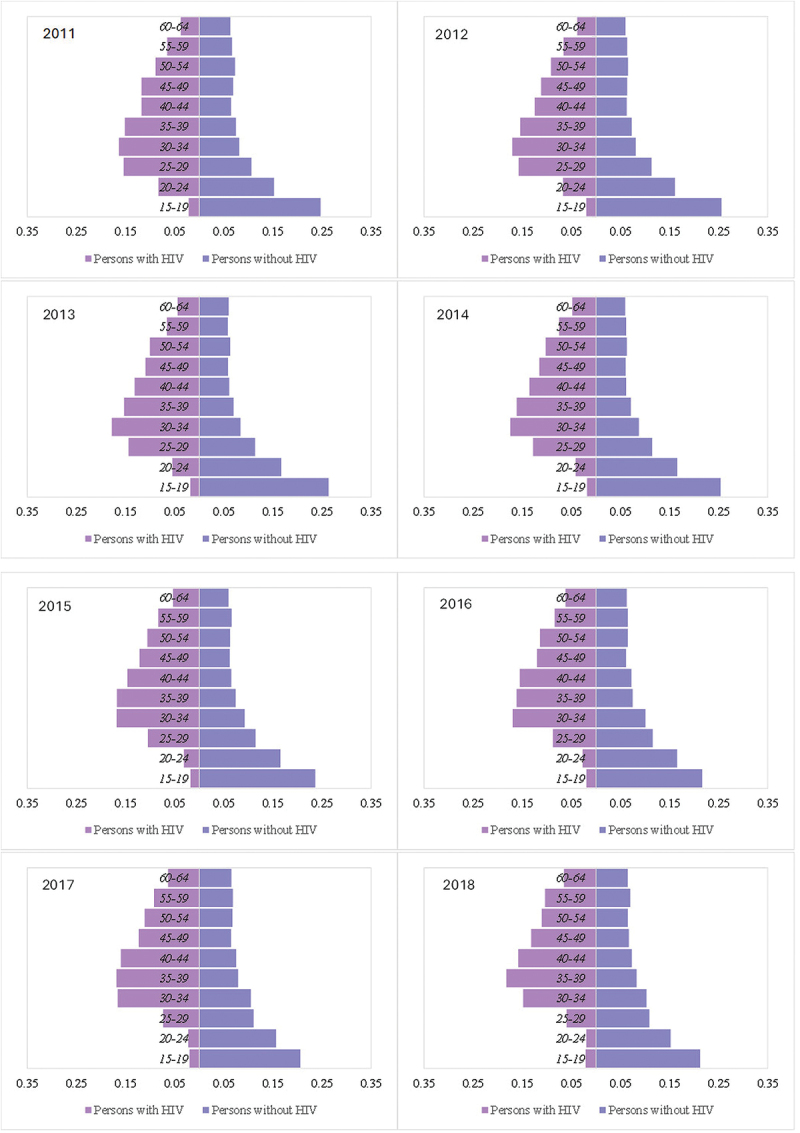
Population pyramids of persons with HIV and persons without HIV in the study population of the Siaya health and demographic surveillance system, from 2011–2018 (population share).

Among PWH, the percentage of persons aged 25–39 years decreased from 46.9% in 2011 to 39.0% in 2018, whilst for PWOH, it increased from 26.3% to 29.5% during the same period (Figure 1). The population share of PWH aged over 50 years old rose from 19.2% in 2011 to 27.9% in 2018. Meanwhile, the population share of PWOH aged over 50 years remained constant at 20.4% in 2011 and 20.0% in 2018 across the same time periods.

### Participant follow-up and causes of death

During follow-up over 2011–2018, 1386 individuals with an HIV test result died, of whom 48.8% (*n* = 676) were PWH. Among PWH, the median age at death peaked in 2017 (IQR: 34–55) and 2018 (IQR: 37–50) at 45 years, whereas among PWOH, it peaked at 53 (IQR: 35–61) years in 2013. The corresponding crude mortality rate for PWH was 22.3 (95% confidence interval [CI]: 20.7–24.0) deaths per 1000 person-years. Among PWOH, the crude mortality rate was 4.0 (3.7–4.3) per 1000 person-years.

Of the 1386 people who died, 1122 (81.0%) had a cause of death ascertained. A detailed description of the grouped causes of death is in Table A8. HIV/AIDS/tuberculosis (319; 58.2%) was the leading cause of death for PWH, followed by NCDs (110; 20.1%), infectious diseases (78; 14.2%), and external/obstetrics causes (29; 5.3%). Among PWOH, NCDs (235; 40.9%) were the leading cause of death, followed by HIV/AIDS/tuberculosis (137; 23.9%), infectious diseases (93; 16.2%), and external/obstetrics causes (93; 16.2%).

**Figure 2.**
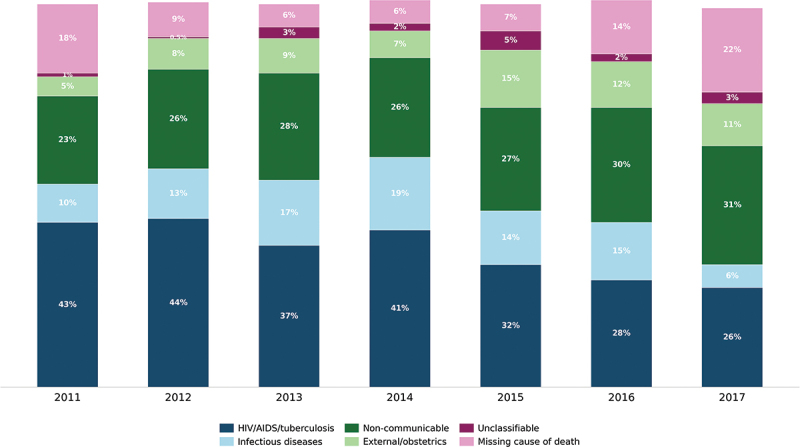
Annual distribution of deaths by cause in the study population of the Siaya health and demographic surveillance system.

By calendar year, HIV/AIDS/tuberculosis was the leading cause of death from 2011 (*n* = 63; 43.2%) to 2015 (*n* = 56; 32.4%) (Figure 2). Post-2015, NCDs became the leading cause of death in 2016 (*n* = 53; 29.6%) and 2017 (*n* = 55; 30.9%). Deaths due to infectious diseases peaked in 2014 at 19.3% (*n* = 37) and then decreased to 6.2% (*n* = 11) in 2017. The percentage of individuals missing a cause of death decreased from 17.8% (*n* = 26) in 2011 to 2014 in 5.7% (*n* = 11) and then increased thereafter from 6.9% (*n* = 12) in 2015 to 22.5% (*n* = 40) in 2017.

### Overall and age-specific trends in all-cause mortality rates by HIV status

**Figure 3.**
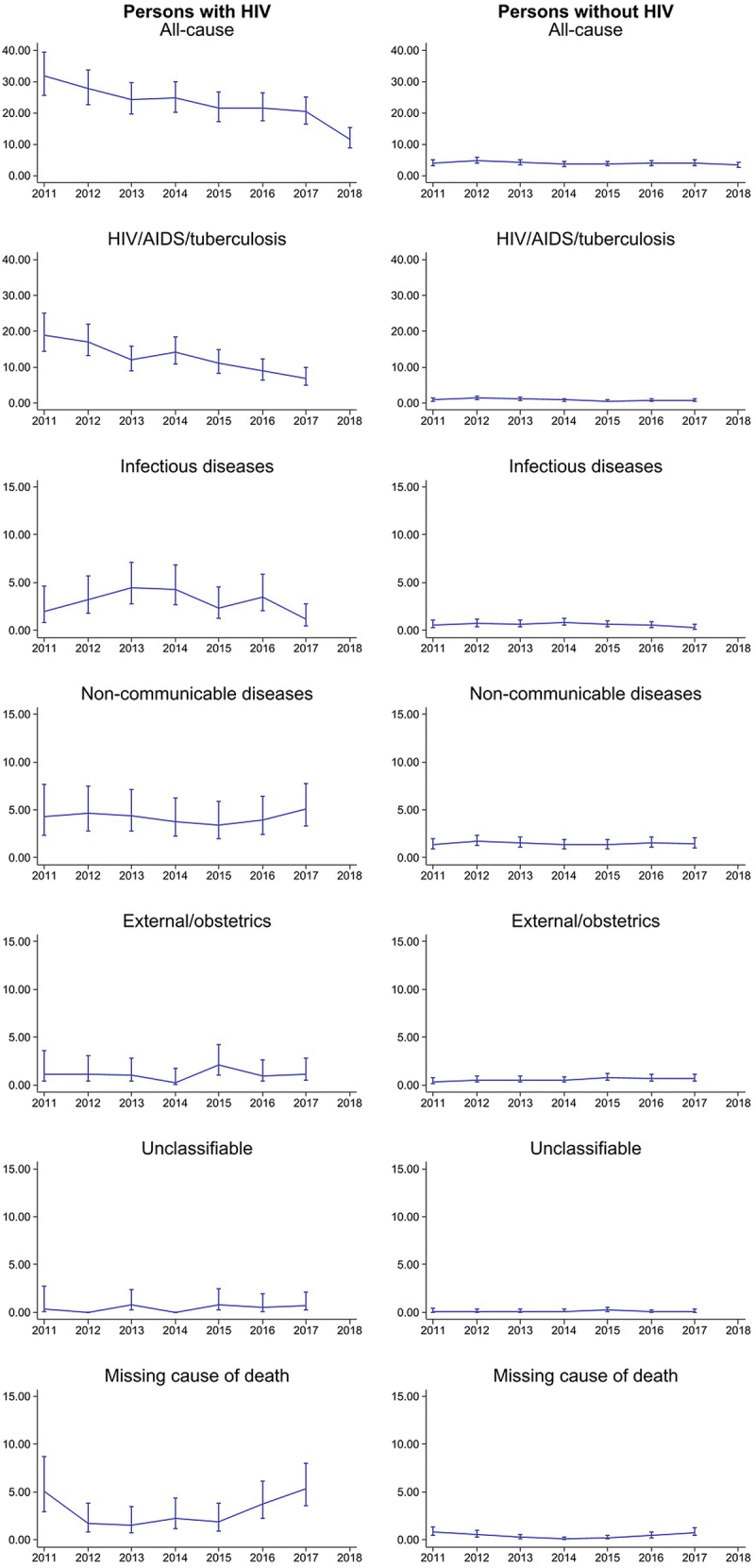
All-cause and cause-specific mortality rates per 1000 person-years by HIV status among the study population in the Siaya health and demographic surveillance system.

Note: Y-axis ranges differ for all-cause and HIV/AIDS/tuberculosis with other causes of death. PWH-people with HIV. PWOH-people without HIV.

Among PWH, the all-cause mortality rate declined from 31.8 (25.6–39.5) deaths per 1000 person-years in 2011 to 11.6 (8.8–15.3) in 2018. Among PWOH, all-cause mortality rates were relatively stable across the years, 3.9 (3.1–5.0) in 2011 and 3.5 (2.8–4.4) deaths per 1000 persons-years in 2018 (Figure 3).

Among PWH, all-cause mortality rates decreased by more than half between 2011 and 2018 in all age-groups. Among those aged 15–34 years, the all-cause mortality rate declined from 23.9 (16.2–35.0) per 1000 person-years in 2011 to 9.7 (5.2–18.1) in 2018. During the same period, mortality rates per 1000 person-years also declined from 29.1 (20.2–41.9) to 12.4 (8.4–18.3) and 54.6 (37.4–79.6) to 11.8 (7.0–20.0), among those aged 35–49 and 50–64 years, respectively.

Among PWOH, age-specific mortality rates were stable in all age-groups across the years. For instance, among those aged 15–34 years mortality rates per 1000 person-years were 2.0 (1.3–3.1) in 2011 and 2.1 (1.4–5.0) in 2018. Among individuals aged 35–49, mortality rates were 2.6 (1.4–5.0) per 1000 person-years and 3.0 (1.8–5.0) during the same period. Similarly, among those aged 50–64 years mortality rates per 1000 person-years were 10.7 (7.8–14.9) in 2011 and 7.9 (5.6–11.1) in 2018.

### Overall and age-specific trends in cause-specific mortality rates by HIV status

Similarly, from 2011 to 2017, HIV/AIDS/tuberculosis mortality rates declined among PWH from 19.0 (14.4–25.1) in 2011 to 7.0 (4.9–9.9) deaths per 1000 persons-years in 2017. Mortality rates due to infectious diseases declined from a peak of 4.4 (2.7–7.1) in 2013 to 1.2 (0.5–2.8) deaths per 1000 person-years in 2017. Meanwhile, mortality rates from NCDs showed a slight upward trend in later years, increasing from 3.7 (2.3–6.2) in 2014 to 5.1 (3.4–7.7) per 1000 person years in 2017. Mortality rates due to external/obstetrics causes were 1.2 (0.4–3.6) in 2011 and 1.2 (0.5–2.8) per 1000 person-years in 2017, with some slight changes in the years between, although with wide confidence intervals. For PWOH, no discernible changes in mortality were observed over time due to deaths caused by HIV/AIDS/tuberculosis, infectious diseases, NCDs, and external/obstetrics causes.

**Figure 4.**
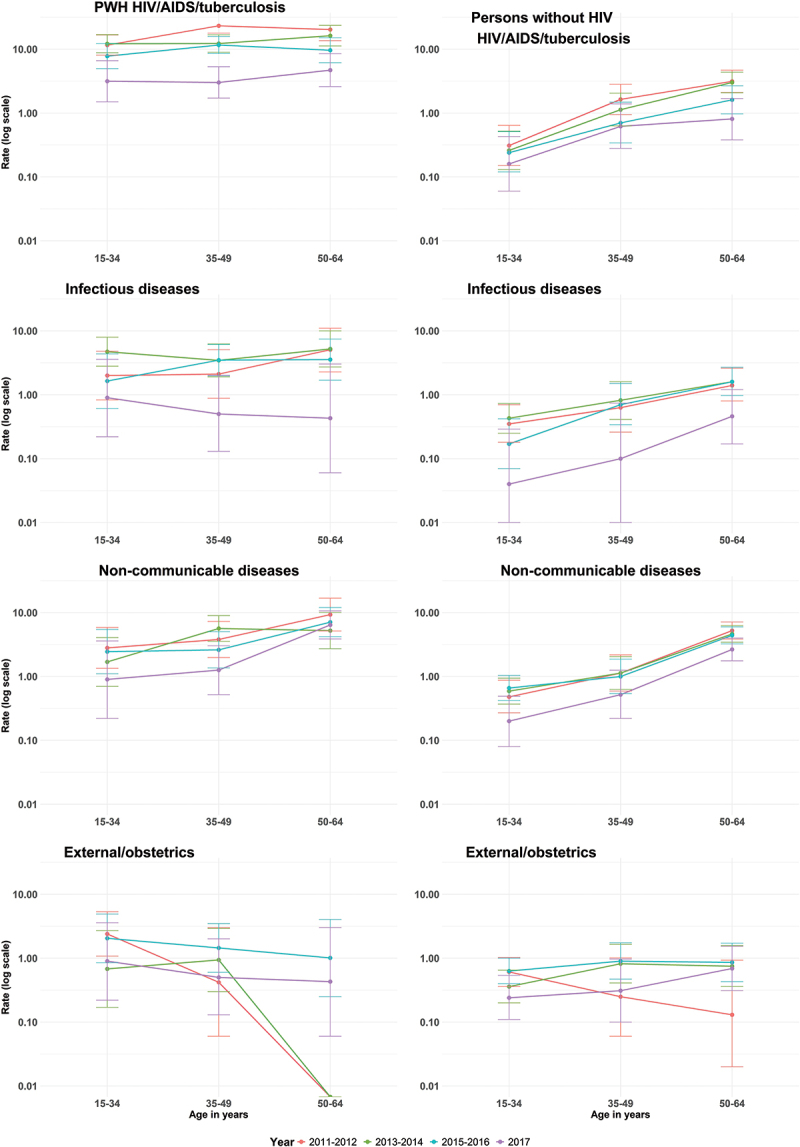
Mortality rates (log scale) by age and cause from 2011 to 2017 in the study population of the Siaya health and demographic surveillance system.

Overall, PWH had higher age- and cause-specific mortality rates than PWOH. Age-and cause-specific mortality rates decreased over time in both groups and were higher among people aged 50–64 than in younger age groups, with the exception of deaths caused by external/obstetrics causes (Figure 4).

### Joinpoint trends in age-standardized mortality rates by HIV status

**Figure 5.**
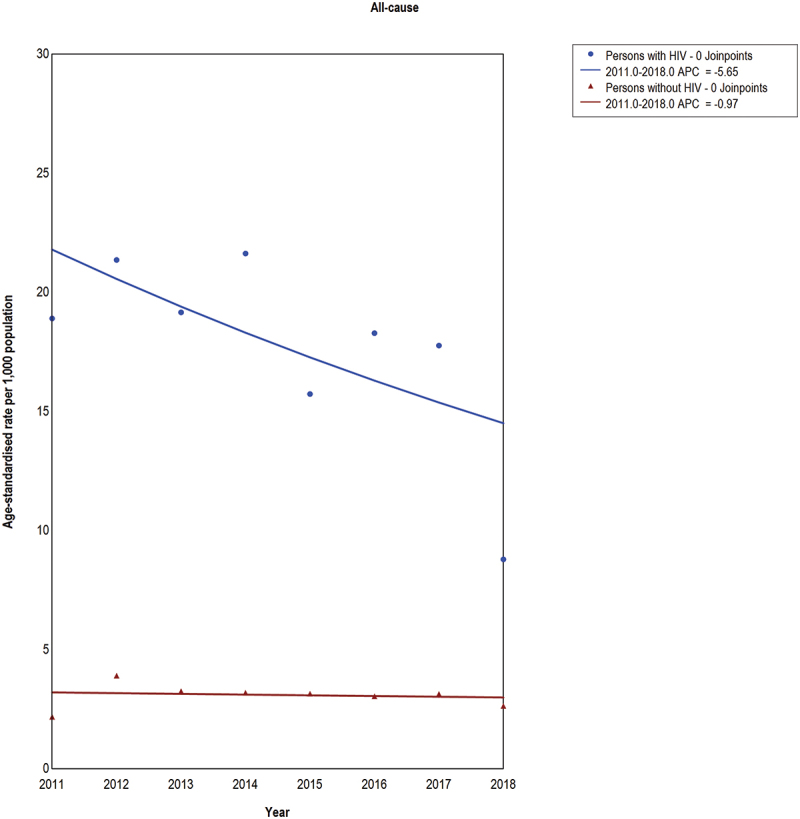
Joinpoint regression analysis of age-standardized all-cause mortality rates per 1000 population by HIV status in the study population of the Siaya health and demographic surveillance system.

Despite mortality reductions, the age-standardized mortality rates remained higher among PWH than PWOH. Between 2011 and 2018, PWH experienced a decrease in age-standardized all-cause mortality, falling from 18.9 (95% CI: 14.5–2.3) deaths per 1000 population in 2011 to 8.8 (95% CI: 5.6–12.0) in 2018 (APC: −5.6 [95% CI: −13.8 to 1.9) (Figure 5 and Table 3). Among PWOH, the age-standardized all-cause mortality rate remained stable during the same period (APC: −1.0 [95% CI: −6.8 to 5.2]), with a rate of 2.2 (95% CI: 1.6–2.7) per 1000 population in 2011 and 2.7 (95% CI: 2.0–3.3) in 2018.

**Table 3.** Joinpoint regression results for all-cause and cause-specific mortality among people with HIV and people without HIV in the study population of the Siaya health and demographic surveillance system.

		Annual percentage change (95% CI)	
Cause of death	Year	People with HIV	People without HIV
All-cause	2011 to 2018	−5.6 (−13.8 to 1.9)	−1.0 (−6.9 to 5.2)
HIV/AIDS/tuberculosis	2011 to 2017	−9.1 (−20.9 to 2.5)	−7.5 (−23.9 to 9.6)
Infectious diseases	2011 to 2014	43.3 (12.9 to 150.1)	25.5 (5.9 to 76.0)
	2014 to 2017	−32.5 (−66.3 to −12.9)	−31.8 (−55.2 to −18.4)
Non-communicable diseases	2011 to 2017	1.0 (−11.8 to 15.0)	2.0 (−5.9 to 10.9)
External/obstetrics causes	2011 to 2017	1.3 (−16.2 to 22.5)	10.0 (−7.1 to 35.4)

Among PWH, HIV/AIDS/tuberculosis deaths decreased annually by 9.1% (95% CI: −20.9 to 2.5). The corresponding age-standardized rates among PWH were 10.5 (95% CI: 7.3–13.7) per 1000 population in 2011 and 5.8 (95% CI: 3.2–8.4) per 1000 population in 2017. Among PWOH, the age-standardized rate of HIV/AIDS/tuberculosis mortality was constant at 0.5 (95% CI: 0.2–0.7) per 1000 population in 2011 and 0.6 (95% CI: 0.3–0.9) per 1000 population in 2017 (APC: −7.5% [95% CI: −23.9 to 9.6]).

Among PWH, the age-standardized rate of infectious disease mortality increased from 1.2 (95% CI: 0.1–2.4) in 2011 to 4.9 (95% CI: 2.3–7.4) deaths per 1000 population in 2014 (APC: 43.3 [95% CI: 12.9 to 150.1]), then decreased to 1.3 (95% CI: 0.0 to 2.6) per 1000 population in 2017 (APC: −32.5 [95% CI: −66.3 to −12.9]). A similar pattern was also observed among PWOH, the age-standardized rate increased from to 0.3 (0.1–0.6) in 2011 to 0.71 (0.4–1.0) in 2014 (APC: 25.5 [95% CI: 5.9 to 76.0]) and decreased to 0.2 (0.04–0.4) per 1000 population in 2017 (APC: −31.8 [95% CI: −55.2 to −18.4]).

Age-standardized rates of NCD deaths remained constant between PWH (APC: 1.0 [95% CI: −11.8 to 15.0]) and PWOH (APC: 2.0 [95% CI: −5.9 to 10.9]). Age-standardized rates of external/obstetrics cause of death remained stable between PWH (APC: 1.3 [95% CI: −16.2 to 22.5]) and PWOH (APC: 10.0 [95% CI: −7.1 to 35.4]).

## Survival modeling of mortality

### Univariable survival model

Between 2011 and 2018, there was a decline in all-cause mortality among PWH (Hazard ratio [HR]: 0.86 [95% CI: 0.81–0.92]) and among PWOH (HR: 0.92 [95% CI: 0.86–0.98]) (Table 4). The full output for the models are presented in Appendix, Table A9.

**Table 4.** Associations of all-cause and cause-specific mortality with calendar year (per 1-year increase), estimated from Cox and competing risks regression models in the study population of the Siaya health and demographic surveillance system from 2011 to 2018.

	Causes of death						
	All-cause	HIV/AIDS/ tuberculosis	Infectious diseases	Non-communicable Diseases	External/obstetrics causes	Unclassifiable	Missing
	Hazard ratio (95% CI)	Subdistribution hazard ratio (95% CI)	Subdistribution hazard ratio (95% CI)	Subdistribution hazard ratio (95% CI)	Subdistribution hazard ratio (95% CI)	Subdistribution hazard ratio (95% CI)	Subdistribution hazard ratio (95% CI)
Persons with HIV Unadjusted	0.86 (0.81–0.92)	0.81 (0.76–0.87)	0.95 (0.85–1.06)	0.93 (0.84–1.03)	1.01 (0.83–1.24)	1.02 (0.80–1.31)	0.90 (0.82–1.00)
Adjusted*	0.88 (0.83–0.93)	0.83 (0.78–0.89)	0.97 (0.87–1.08)	0.96 (0.87–1.06)	1.03 (0.84–1.26)	1.07 (0.85–1.35)	0.91 (0.82–1.01)
Persons without HIV Unadjusted	0.92 (0.86–0.98)	0.80 (0.70–0.91)	0.73 (0.64–0.83)	0.93 (0.84–1.04)	0.94 (0.83–1.07)	0.94 (0.73–1.23)	0.82 (0.63–1.06)
Adjusted*	0.96 (0.90–1.02)	0.85 (0.75–0.96)	0.77 (0.68–0.87)	0.99 (0.89–1.09)	0.97 (0.85–1.09)	0.98 (0.77–1.26)	0.87 (0.68–1.10)

95% CI: 95% Confidence Interval; *Variables for which the models were adjusted were age and sex.

Among PWH, the hazard of dying from HIV/AIDS/tuberculosis declined by 19% per-one year increase (sHR: 0.81 [95% CI: 0.76–0.87]). Similarly, the HIV/AIDS/tuberculosis mortality subdistribution hazard declined over time among PWOH (sHR: 0.80 [95% CI: 0.70–0.91]). Meanwhile, the subdistribution hazard ratio of the annual change of dying due to infectious diseases showed no evidence of change among PWH (sHR: 0.95 [95% CI: 0.85–1.06]) but a decreasing annual trend among PWOH (sHR: 0.73 [95% CI: 0.64–0.83]). There was no strong evidence of the hazard of dying from NCDs changing over time in either PWH (sHR: 0.93 [95% CI: 0.84–1.03]) or PWOH (sHR: 0.93 [95% CI: 0.84–1.04]). Similarly, there was no evidence of annual changes in the hazard of dying from external/obstetrics causes among PWH (sHR: 1.01 [95% CI: 0.83–1.24]) and PWOH (sHR: 0.94 [95% CI: 0.83–1.07]). Among PWH with missing cause-of-death information, the hazard of dying decreased across the years. However, a time-varying sHR of 1.07 (95% CI: 1.03–1.10) indicated that the relative hazard of missing cause of death information increased as the calendar year progressed. Among PWOH, the hazard of death did not change among individuals for whom the cause of death was missing (sHR: 0.82 [95% CI: 0.63–1.06]).

### Multivariable survival model

Between 2011 and 2018, there was a decline in the hazard of death among PWH (adjusted hazard ratio [aHR]: 0.88 [95% CI: 0.83–0.93]). Among PWOH, no strong evidence of a change in the annual hazard of mortality was observed (aHR: 0.96 [95% CI: 0.90–1.02]). In the competing risk regression model, the subdistribution and hazard ratios of the different causes of death in the multivariable models were similar to those in the univariable models (Table 4).

### Interaction of HIV status and calendar year

In the models containing interaction terms between HIV status and year, there was no strong evidence of a change in all-cause mortality among PWOH over time (aHR: 0.96 [95% CI: 0.91–1.01]) (Table A10). However, the interaction between HIV status and year indicates that the hazard of all-cause mortality decreased per-one year increase for PWH (aHR: 0.91 [95% CI: 0.87–0.96]). Meanwhile, the effect of per-one year increase on the sub-hazards of dying from HIV/AIDS/tuberculosis did not differ by HIV status, indicating that the decline in mortality was comparable between PWH and PWOH (adjusted sHR: 0.94 [95% CI: 0.85–1.04]). Similarly, mortality patterns from infectious diseases, NCDs, external/obstetrics causes, unclassifiable causes, and those missing cause of death information in PWH were comparable to those observed in PWOH.

### Sensitivity analysis

A sensitivity analysis altering the assumed person-time spent as HIV-negative following a negative HIV test from 5 years to 2 years, produced minimal variation in the mortality hazard ratios (Appendix Table A11).

## Discussion

This analysis examined demographic changes and mortality trends among individuals aged between 15 and 64 years, with and without HIV in Siaya HDSS from 2011 to 2018. The median age of PWH increased from 37 (IQR: 29–47) to 42 (IQR: 35–51), while that of PWOH rose from 29 (IQR: 20–46) to 31 (IQR: 20–46) years. In analyses of crude and age-standardized mortality rates, PWH experienced reductions in all-cause mortality, whereas all-cause mortality among PWOH stayed mostly stable. For PWH, crude cause-specific mortality rates due to HIV/AIDS/tuberculosis and infectious diseases declined over time, while NCD-related mortality increased and other causes of death remained constant. Meanwhile, for PWOH, crude cause-specific mortality rates remained stable across the period. Among PWH, the unadjusted HIV/AIDS/tuberculosis mortality hazard decreased over time, but this was not seen for mortality due to NCDs or other causes. PWOH saw reductions in unadjusted mortality hazards for HIV/AIDS/tuberculosis and infectious diseases, but not NCDs.

## Trends in all-cause mortality and demographic changes by HIV status

Our analysis shows that the proportion PWH aged 25–39 years has decreased, while the older age groups have grown. The analysis also found a threefold reduction in all-cause mortality within the 25–39 age group in the Gem region of the HDSS, likely due to expanded ART access over the past decade. This decline in mortality has allowed younger cohorts to live longer and transition into older age groups, leading to an ageing population with HIV.

Among PWOH, age-standardised all-cause mortality rates remained stable, while the unadjusted hazard of dying decreased each year. This suggests that improvements in mortality may stem from an increasing population of younger, healthier individuals over time. These demographic changes are likely a result of the decline in HIV incidence among younger age groups, which can be attributed to effective HIV prevention measures and reduced rates of mother-to-child HIV transmission [39,40]. Consequently, fewer young people are testing positive for HIV. Additionally, the decrease in HIV incidence among those aged 15–24 has contributed to a larger share of older individuals among PWH, as fewer younger individuals with HIV are available to replace those PWH ageing into older age groups [39]. As a result, the HIV-negative population tends to have a younger age profile compared to the ageing HIV-positive population.

## Epidemiological transition in Siaya HDSS

The cause-specific mortality patterns among people with and without HIV in our analysis exemplify Abdel Omran’s classic epidemiological transition model, in which societies progress from an ‘age of pestilence and famine’ dominated by infectious diseases and high mortality, to an ‘age of receding pandemics’ in which infectious diseases decline, to an ‘age of degenerative and man-made diseases’ where NCDs predominate [41].

## Additional comparisons with other literature on mortality trends among persons with and without HIV

Consistent with our results, other studies also found that all-cause mortality rates among PWOH have been stable across the years in Siaya HDSS [10,42]. Additionally, studies conducted in Zimbabwe and Uganda observed similar reductions in mortality among PWH between 2000 and 2010, while mortality among PWOH remained stable [42]. The stable mortality rate among PWOH likely results from the predominantly young population in these settings, as younger individuals generally have a lower risk of death. For instance, in our analysis, the median age at death reached 53 years, suggesting that mortality among PWOH is being postponed in these communities.

Mortality levels among PWH are still higher than PWOH even after the increased availability of ART access in this community [7]. The high mortality rates among PWH may be a result of moderate ART uptake in this community as only 61% of the adults with HIV were on ART as of 2017 [43]. The high mortality rates among PWH compared to PWOH may also be driven by challenges related to starting ART and suboptimal adherence to ART. Information and educational programmes addressing the sub-optimal ART uptake among individuals diagnosed with HIV could also have long-term impacts on attitudes towards taking ART in this community. In a previous study in this region, men had higher mortality hazards of dying than women, potentially due to their lower uptake in HIV testing and treatment [44]. It may well be that the enrolment of women into ART programs was more effective through their integration with antenatal and reproductive health services.

## Cause-specific mortality

### HIV/AIDS and tuberculosis

The decline in HIV/AIDS and tuberculosis mortality has accounted for much of the decrease in all-cause mortality among PWH in Siaya, aligning with the implementation and expansion of ART in Siaya HDSS [7,10]. Moreover, all patients visiting outpatient departments are also screened for tuberculosis, which may explain the decline in tuberculosis mortality among PWOH [45]. Siaya County also benefits from an effective tuberculosis programme, achieving a cure rate of over 80% [45]. The decline in HIV/AIDS and tuberculosis mortality among persons with and without HIV shows the successful implementation and integration of HIV/AIDS and tuberculosis programs, offering adaptation examples.

Additionally, the decline in HIV/AIDS/tuberculosis mortality in this region may be attributed to free access to HIV prevention, testing, and treatment [46]. There are 13 health facilities in Gem region, Siaya HDSS that provide HIV care, with a median distance of 1 km to the nearest one [27]. To further decongest health facilities and improve ART adherence, differentiated HIV care services such as multi-month dispensation of ART were implemented post-2016 [47].

To achieve mortality rates among PWH that are comparable to those of PWOH, it may be necessary to meet the 95–95–95 UNAIDS target to further enhance survival rates. In Siaya County, the UNAIDS target in 2016 was 54–91-64, meaning that 54% of PWH were aware of their status, 91% of those diagnosed were receiving ART, and 64% of those on ART had achieved viral suppression [48,49]. By 2018, these UNAIDS indicators in Siaya County had improved to 86–93–93 [3].

### Non-communicable diseases

The percentage of PWH aged over 50 years increased by nearly 10% points between 2011 and 2018 (from 19% to 28%), while there was no change among PWOH (20%). The median age at death among PWH also increased from 38 years in 2012 to 45 in 2018. Our analysis showed an upward trend in crude NCD-related mortality rates among PWH, implying that NCD deaths are increasing over time as PWH is living longer. Since Dolutegravir was introduced as the first-line ART regimen in Kenya in 2018, and some evidence suggests it may raise the risk of hypertension and obesity [50,51], it is possible that age-related NCDs could increase over time among PWH, which may lead to increases in cardiovascular mortality.

A modeling study also projected that the percentage of PWH in Kenya with NCDs would increase from 60% in 2018 to 70% in 2035 [52]. Nationally, NCD deaths continue to rise: the proportion of deaths that were due to NCDs in Kenya increased from 27% in 2014 to 39% in 2020 [53]; in our analysis NCD-related deaths among persons with and without HIV rose from 23% in 2011 to 31% in 2017. A study in a nearby Kenyan county with a HIV prevalence of 17.5% found that, in 2019, NCDs were the leading cause of death among adults, followed by AIDS [3,54].

On the other hand, we found that the hazard of dying from NCDs among PWH was stable across the years because these individuals ageing with HIV may have comprised of healthier individuals, a concept referred to as the survivor effect [55]. They may have a healthier lifestyle and a higher use of health services, closer monitoring of NCDs and a better understanding of NCD prevention. The stable hazards of dying from NCDs, despite the ageing of the PWH population, suggest that these interventions may have been successful.

### Infectious diseases

The hazard of infectious disease mortality declined over time among PWOH, implying that public health interventions may have been effective in reducing premature deaths from preventable diseases in this community. However, the lack of decline observed among PWHmay be related to the unique challenges caused by HIV, such as lowered immunity and the HIV infection itself [56], increasing the risk of dying from these infectious diseases and not the differential application of these interventions. Individuals with HIV engaged in care are generally given priority in insecticide treated bed-nets distribution and water filters [57], provision of sodium hypochlorite for treating drinking water [7], and some ART programmes provide nutritional support [58]. The observed increase in infectious disease mortality rates from 2011 to 2014 within the HDSS may reflect a data artefact.

### External/obstetrics causes

In our analysis, we found that deaths from external/obstetric causes did not increase over time. There was variability in trends based on different age categories and HIV status, which may be attributed to the limited number of individuals who died from these causes within the HDSS.

### Strengths and limitations

This study utilizes primary population-based data from a population that has been under rigorous surveillance for several years, and the results are likely to translate to other sub-Saharan contexts. Using population-based mortality data also reduces the likelihood of loss to follow-up compared to ART programs, implying that our mortality trend estimates are unlikely to be biased downward [59]. Furthermore, out-migration was relatively stable during the study period, limiting selection bias.

This analysis has some limitations. Firstly, we lacked individual data on ART status. Thus, we could not stratify mortality data by ART status to evaluate the impact of ART on cause-specific mortality patterns. Secondly, we only studied mortality trends up until 2018, so further data would be required to understand what has happened since then. Thirdly, we did not have verbal autopsy data for 2018. Fourthly, cause-specific mortality trends may have been underestimated by the higher percentage of missing cause of death data in both earlier (2011; 18%) and later (2017; 22%) years. By design, verbal autopsy has limitations. For instance, the ability of the InterVA-4 to assign a cause of death in the NCDs category may have been imprecise based on the reported signs and symptoms the deceased presented with. In the NCDs cause of death category, acute abdomen was the leading cause of death followed by digestive neoplasm. This implies that symptoms like abdominal pain, which can be linked to multiple conditions, were probably classified as digestive neoplasms or acute abdomen, which could have been a misclassification. To attempt to adjust for this bias, our analysis included broader cause of death categories. Misclassification of cause of death could have occurred among individuals who had been taking ART for an extended period as non-AIDS related deaths may present with nonspecific symptoms, potentially distorting observed cause-specific mortality trends in older age groups. Lastly, the effective sample size was relatively small for a study of cause-specific mortality, and the uncertainty around the estimates was inevitably large.

## Conclusion

This analysis assessed how cause-specific mortality has evolved among people aged 15–64 with and without HIV, taking into account the impact of demographic changes on this population. All-cause mortality among PWH in Siaya HDSS has decreased, and they are living longer. However, this increase in longevity raises the risk of age-related conditions that will require chronic management, similar to HIV. While mortality from HIV/AIDS/tuberculosis among PWH has decreased, it remains the leading cause of death in this group. This situation underscores the necessity to more effectively address the UNAIDS 95–95–95 targets for this community.

## Recommendations and policy implications

Given the rising burden of NCDs among PWH globally, the most recent global policy from the United Nations High-Level Meeting on HIV/AIDS in mid-2021 set a goal to provide 90% of PWH with integrated healthcare that includes HIV, mental health, and both communicable and non-communicable diseases [60]. Integrating these health services can lower costs in healthcare facilities, particularly in staffing, and promote the sustainable delivery of HIV-related and other health services [61]. In Kenya, policies related to the integration of HIV with NCDs as part of standard care were included in the 2016 ART guidelines [62]. This policy contains details on screening and management of cervical cancer, hypertension, diabetes, cholesterol, kidney disease and mental health conditions. However, the integration of health care services is also context/region-specific, and more research is required to compare patient outcomes including HIV/hypertension/diabetes control, adherence, and retention in care with non-integrated models [60].

In the Siaya HDSS, PWH received HIV treatment services integrated into the primary health care system (health centers/dispensaries) [63]. The advantage of integrating HIV treatment within primary health care is that PWH receive both HIV-related and non-HIV-related health services in the same consultation. This may ensure the delivery of seamless healthcare services to the ageing HIV population with comorbidities. However, some health system-related challenges include the lack of specialized diagnostic equipment in these health centers/dispensaries; thus, some cases may need to be referred to other health facilities [63]. This suggests that there will be a need to strengthen the referral mechanisms, as some PWH may not reach the next level of care. Having a referral coordinator in these health centers/dispensaries to assist referral cases reach the recommended specialist could be a potential strategy [64].

## Supplementary Material

Supplemental Material

## Data Availability

Data used in this manuscript originated from the Siaya Health Demographic Surveillance System (HDSS), Kenya. Requests for permission to use the data from Siaya HDSS should be directed to Daniel Kwaro; email: dkwaro@kemricdc.org.

## References

[1] Central Bureau of Statistics - CBS/Kenya and Ministry of Health - MOH/Kenya. ORC MRacro. Kenya demographic and health survey 2003. Calverton (MD), USA: CBS, MOH, and ORC Macro; 2004.

[2] Maina WK, Kim AA, Rutherford GW, et al. Kenya AIDS indicator surveys 2007 and 2012: implications for public health policies for HIV prevention and treatment. J Acquir Immune Defic Syndr. 2014;66:S130–18. 24732817 10.1097/QAI.0000000000000123 PMC4784700

[3] National AIDS & STI Control Programme. Kenya population-based HIV impact assessment KENPHIA 2018. Nairobi, Kenya: Ministry of Health; 2018.

[4] Young PW, Musingila P, Kingwara L, et al. HIV incidence, recent HIV infection, and associated factors, Kenya, 2007–2018. AIDS Res Hum Retroviruses. 2023;39:57–67. 36401361 10.1089/aid.2022.0054 PMC9942172

[5] Achoki T, Miller-Petrie MK, Glenn SD, et al. Health disparities across the counties of Kenya and implications for policy makers, 1990–2016: a systematic analysis for the Global Burden of Disease Study 2016. Lancet Glob Health. 2019;7:e81–e95. 30482677 10.1016/S2214-109X(18)30472-8 PMC6293072

[6] Kenya National Bureau of Statistics. Economic survey 2022. Nairobi, Kenya: Kenya National Bureau of Statistics; 2022.

[7] Gargano JW, Laserson K, Muttai H, et al. The adult population impact of HIV care and antiretroviral therapy in a resource poor setting, 2003–2008. AIDS. 2012;26:1545–1554. doi: 10.1097/QAD.0b013e328353b7b9 22441254

[8] United Nations Office for the Coordination of Humanitarian Affairs. Free ARVs a step in the right direction, but much more needed. The New Humanitarian; 2006.

[9] Ambia J, Renju J, Wringe A, et al. From policy to practice: exploring the implementation of antiretroviral therapy access and retention policies between 2013 and 2016 in six sub-Saharan African countries. BMC Health Serv Res. 2017;17:758. doi: 10.1186/s12913-017-2678-1 29162065 PMC5698969

[10] Otieno G, Whiteside YO, Achia T, et al. Decreased HIV-associated mortality rates during scale-up of antiretroviral therapy, 2011–2016. AIDS. 2019;33:2423–2430. doi: 10.1097/QAD.0000000000002374 31764107

[11] World Health Organization. Antiretroviral therapy for HIV infection in adults and adolescents: recommendations for a public health approach - 2010 revision. 2010 rev ed. Geneva: World Health Organization; 2010. 23741771

[12] World Health Organization. Consolidated guidelines on the use of antiretroviral drugs for treating and preventing HIV infection: summary of key features and recommendations, June 2013. Geneva, Switzerland: World Health Organization; 2013.

[13] World Health Organization. Consolidated guidelines on the use of antiretroviral drugs for treating and preventing HIV infection: recommendations for a public health approach. 2nd ed. Geneva: World Health Organization; 2016. 27466667

[14] Borgdorff MW, Kwaro D, Obor D, et al. HIV incidence in Western Kenya during scale-up of antiretroviral therapy and voluntary medical male circumcision: a population-based cohort analysis. Lancet HIV. 2018;5:e241–e9. 29650451 10.1016/S2352-3018(18)30025-0

[15] Phillips-Howard PA, Laserson KF, Amek N, et al. Deaths ascribed to non-communicable diseases among rural Kenyan adults are proportionately increasing: evidence from a health and demographic surveillance system, 2003–2010. PLoS One. 2014;9:e114010. doi: 10.1371/journal.pone.0114010 25426945 PMC4245262

[16] Odhiambo FO, Laserson KF, Sewe M, et al. Profile: the KEMRI/CDC health and demographic surveillance system–Western Kenya. Int J Epidemiol. 2012;41:977–987. 22933646 10.1093/ije/dys108 PMC12083774

[17] Amek N, Vounatsou P, Obonyo B, et al. Using health and demographic surveillance system (HDSS) data to analyze geographical distribution of socio-economic status; an experience from KEMRI/CDC HDSS. Acta Trop. 2015;144:24–30. doi: 10.1016/j.actatropica.2015.01.006 25602533

[18] Kenya National Bureau of Statistics. Basic report on well being in Kenya. Based on the 2015/2016 Kenya integrated household budget survey (KIHBS). Nairobi, Kenya: Kenya National Bureau of Statistics; 2018.

[19] Kenya National Bureau of Statistics. The Kenya poverty report. Based on the 2022 Kenya continuous household survey. Nairobi, Kenya: Kenya National Bureau of Statistics; 2024.

[20] Slaymaker E, McLean E, Wringe A, et al. The network for analysing longitudinal population-based HIV/AIDS data on Africa (ALPHA): data on mortality, by HIV status and stage on the HIV care continuum, among the general population in seven longitudinal studies between 1989 and 2014. Gates Open Res. 2017;1:4. doi: 10.12688/gatesopenres.12753.1 29528045 PMC5841576

[21] Eilerts-Spinelli H, Prieto JR, Ambia J, et al. Evaluating pregnancy reporting in Siaya Health and Demographic Surveillance System through record linkage with ANC clinics. Int J Popul Data Sci. 2022;7:1762. doi: 10.23889/ijpds.v7i4.1762 37181491 PMC10167572

[22] INDEPTH Network. Indepth standardized verbal autopsy questionnaire 2002 [updated 2003 Aug]. Available from: https://www.indepth-network.org/resources/indepth-standardized-verbal-autopsy-questionnaire

[23] Byass P, Calvert C, Miiro-Nakiyingi J, et al. InterVA-4 as a public health tool for measuring HIV/AIDS mortality: a validation study from five African countries. Glob Health Action. 2013;6:22448. doi: 10.3402/gha.v6i0.22448 24138838 PMC3800746

[24] Herbst AJ, Mafojane T, Newell ML. Verbal autopsy-based cause-specific mortality trends in rural KwaZulu-Natal, South Africa, 2000–2009. Popul Health Metr. 2011;9:47. doi: 10.1186/1478-7954-9-47 21819602 PMC3160940

[25] Byass P, Chandramohan D, Clark SJ, et al. Strengthening standardised interpretation of verbal autopsy data: the new InterVA-4 tool. Glob Health Action. 2012;5:1–8. doi: 10.3402/gha.v5i0.19281 PMC3433652 22944365

[26] Glynn JR, Calvert C, Price A, et al. Measuring causes of adult mortality in rural northern Malawi over a decade of change. Glob Health Action. 2014;7:23621. doi: 10.3402/gha.v7.23621 24802384 PMC4007026

[27] Ambia J, Romero-Prieto JE, Kwaro D, et al. Comparison of programmatic data from antenatal clinics with population-based HIV prevalence estimates in the era of universal test and treat in western Kenya. PLoS One. 2023;18:e0287626. doi: 10.1371/journal.pone.0287626 37363902 PMC10292704

[28] Gust DA, Pan Y, Otieno F, et al. Factors associated with physical violence by a sexual partner among girls and women in rural Kenya. J Glob Health. 2017;7:020406. doi: 10.7189/jogh.07.020406 28959439 PMC5609512

[29] Ginsburg C, Bocquier P, Béguy D, et al. Association between internal migration and epidemic dynamics: an analysis of cause-specific mortality in Kenya and South Africa using health and demographic surveillance data. BMC Public Health. 2018;18:918. doi: 10.1186/s12889-018-5851-5 30049267 PMC6062880

[30] Kimotho J, Ng’ang’a Z, Nyairo E, et al. Laboratory evaluation of the validity of the current HIV testing algorithm in Kenya. Am J Intern Med. 2015;3:23–28. doi: 10.11648/j.ajim.20150301.14

[31] Luseno WK, Field SH, Iritani BJ, et al. Does venue of HIV testing and results disclosure in the context of a Research study affect adolescent Health and behavior? Results from a study in western Kenya. Int J Environ Res Public Health. 2022;19:3249. doi: 10.3390/ijerph19063249 35328936 PMC8953200

[32] Joinpoint Regression Program. Version 5.3.0 - November 2024; Statistical Methodology and Applications Branch, Surveillance Research Program, National Cancer Institute; 2024.

[33] Sankoh O, Sharrow D, Herbst K, et al. The indepth standard population for low- and middle-income countries, 2013. Glob Health Action. 2014;7:23286. doi: 10.3402/gha.v7.23286 24679543 PMC3969509

[34] Schwarz G. Estimating the dimension of a model. Ann Stat. 1978;6:461–464. doi: 10.1214/aos/1176344136

[35] Kim HJ, Fay MP, Feuer EJ, et al. Permutation tests for joinpoint regression with applications to cancer rates. Stat Med. 2000;19:335–351. doi: 10.1002/(SICI)1097-0258(20000215)19:3<335::AID-SIM336>3.0.CO;2-Z 10649300

[36] Robinson CD, Schumacker R, editors. Interaction effects: centering, variance inflation factor, and interpretation issues. Birmingham, USA: University of Alabama; 2009.

[37] Fine JP, Gray RJ. A proportional hazards model for the subdistribution of a competing risk. J Am Stat Assoc. 1999;94:496–509. doi: 10.1080/01621459.1999.10474144

[38] StataCorp. Stata statistical software: Release 18. College Station (TX): StataCorp LLC; 2023.

[39] Birdthistle I, Kwaro D, Shahmanesh M, et al. Evaluating the impact of dreams on HIV incidence among adolescent girls and young women: a population-based cohort study in Kenya and South Africa. PLoS Med. 2021;18:e1003837. doi: 10.1371/journal.pmed.1003837 34695112 PMC8880902

[40] Mwau M, Bwana P, Kithinji L, et al. Mother-to-child transmission of HIV in Kenya: a cross-sectional analysis of the national database over nine years. PLoS One. 2017;12:e0183860. doi: 10.1371/journal.pone.0183860 28850581 PMC5574578

[41] Omran AR. The epidemiologic transition: a theory of the epidemiology of population change. 1971. Milbank Q. 2005;83:731–757. doi: 10.1111/j.1468-0009.2005.00398.x 16279965 PMC2690264

[42] Reniers G, Slaymaker E, Nakiyingi-Miiro J, et al. Mortality trends in the era of antiretroviral therapy: evidence from the network for analysing longitudinal population based HIV/AIDS data on Africa (ALPHA). AIDS. 2014;28:S533–42. doi: 10.1097/QAD.0000000000000496 25406756 PMC4251911

[43] National AIDS Control Council. Siaya County report on the HIV implementing partners online reporting system (HIPORS) for the financial year 2016/2017. Nairobi, Kenya: Ministry of Health; 2018.

[44] Ackers ML, Hightower A, Obor D, et al. Health care utilization and access to human immunodeficiency virus (HIV) testing and care and treatment services in a rural area with high HIV prevalence, Nyanza Province, Kenya, 2007. Am J Trop Med Hyg. 2014;90:224–233. doi: 10.4269/ajtmh.13-0181 24323517 PMC3919222

[45] Center for Health Solutions (CHS). Siaya County government. Ministry of Health and US Centers for Disease Control and Prevention (CDC) Kenya. Analysis of tuberculosis case finding strategies, tuberculosis preventive therapy uptake, and tuberculosis treatment outcomes among clients in general outpatient setting and people living with HIV in Siaya County, Kenya. Siaya, Kenya; 2021.

[46] Olney JJ, Braitstein P, Eaton JW, et al. Evaluating strategies to improve HIV care outcomes in Kenya: a modelling study. Lancet HIV. 2016;3:e592–e600. doi: 10.1016/S2352-3018(16)30120-5 27771231 PMC5121132

[47] National AIDS and STI Control Programme. Improving the quality and efficiency of Health services in Kenya: a practical handb HIV managers service providers differentiated care: natl AIDS STI Control Programme (NASCOP). 2016.

[48] Frescura L, Godfrey-Faussett P, Feizzadeh AA, et al. Achieving the 95 95 95 targets for all: a pathway to ending AIDS. PLoS One. 2022;17:e0272405. doi: 10.1371/journal.pone.0272405 35925943 PMC9352102

[49] Department of Health and Sanitation – Siaya and National AIDS Control Council. Siaya County HIV and AIDS strategic plan (2015/16 – 2018/2019). Siaya, Kenya: Department of Health and Sanitation; 2016.

[50] Ministry of Health Kenya. Guidelines on use of antiretroviral drugs for treating and preventing HIV in Kenya. Nairobi, Kenya: Ministry of Health; 2018.

[51] Shamu T, Egger M, Mudzviti T, et al. Body weight and blood pressure changes on dolutegravir-, efavirenz- or atazanavir-based antiretroviral therapy in Zimbabwe: a longitudinal study. J Int AIDS Soc. 2024;27:e26216. doi: 10.1002/jia2.26216 38332525 PMC10853595

[52] Smit M, Perez-Guzman PN, Mutai KK, et al. Mapping the current and future noncommunicable disease burden in Kenya by human immunodeficiency virus status: a modeling study. Clin Infect Dis. 2020;71:1864–1873. doi: 10.1093/cid/ciz1103 31734688 PMC8240998

[53] Ministry of Health. National strategic plan for the prevention and control of non-communicable diseases | 2021 – 2026. Nairobi, Kenya: Department of Non-communicable Diseases; 2021.

[54] Waruru A, Onyango D, Nyagah L, et al. Leading causes of death and high mortality rates in an HIV endemic setting (Kisumu County, Kenya, 2019). PLoS One. 2022;17:e0261162. doi: 10.1371/journal.pone.0261162 35051186 PMC8775329

[55] Arrighi HM, Hertz-Picciotto I. The evolving concept of the healthy worker survivor effect. Epidemiology. 1994;5:189–196. doi: 10.1097/00001648-199403000-00009 8172994

[56] McDougal JS, Nicholson JKA, Mawle A. Effects of HIV infection on the immune system. In: Madhok R, Forbes CD Evatt BL, editors. Blood, blood products — and AIDS —. Boston (MA): Springer US; 1987. p. 51–88.

[57] Walson JL, Sangaré LR, Singa BO, et al. Evaluation of impact of long-lasting insecticide-treated bed nets and point-of-use water filters on HIV-1 disease progression in Kenya. AIDS. 2013;27:1493–1501. doi: 10.1097/QAD.0b013e32835ecba9 23324658

[58] USAID Kenya. Nutrition and HIV program. Nairobi, Kenya: FH360; 2014.

[59] Rachlis B, Ochieng D, Geng E, et al. Implementation and operational research: evaluating outcomes of patients lost to follow-up in a large comprehensive care treatment program in Western Kenya. J Acquir Immune Defic Syndr. 2015;68:e46–55. doi: 10.1097/QAI.0000000000000492 25692336 PMC4348019

[60] United Nations General Assembly. Political declaration on HIV and AIDS: ending inequalities and getting on track to end AIDS by 2030: resolution/adopted by the General Assembly. Geneva, Switzerland: UNAIDS; 2021 June 9.

[61] Bulstra CA, Hontelez JAC, Otto M, et al. Integrating HIV services and other health services: a systematic review and meta-analysis. PLoS Med. 2021;18:e1003836. doi: 10.1371/journal.pmed.1003836 34752477 PMC8577772

[62] Ministry of Health National AIDS & STI Control Programme (NASCOP). Guidelines on use of antiretroviral drugs for treating and preventing HIV infections in Kenya: 2016 edition. Nairobi, Kenya: NASCOP; 2016 01 Apr 2025.

[63] Duffy M, Ojikutu B, Andrian S, et al. Non-communicable diseases and HIV care and treatment: models of integrated service delivery. Trop Med Int Health. 2017;22:926–937. doi: 10.1111/tmi.12901 28544500

[64] Watt N, Sigfrid L, Legido-Quigley H, et al. Health systems facilitators and barriers to the integration of HIV and chronic disease services: a systematic review. Health Policy Plan. 2017;32:Siv13–iv26. 10.1093/heapol/czw149 PMC5886067 28666336

