# Supplementary material for: All-cause and cause-specific mortality trends among people with and without HIV in the Siaya health and demographic surveillance system, Kenya, 2011–2018
Source: Glob Health Action. 2026 Mar 5;19(1):2640299. doi: 10.1080/16549716.2026.2640299 (PMC12964471; doi:10.1080/16549716.2026.2640299)
Supplement: Supplemental Material [file ZGHA_A_2640299_SM4574.docx]

**All-cause and cause-specific mortality trends among people with and without HIV in the Siaya Health and Demographic Surveillance System, Kenya, 2011-2018**

**Appendix**

**Description of population residing within Gem region of the Siaya** **health and demographic surveillance system**

The mid-year population remained relatively constant throughout the years in both the Siaya HDSS and the Gem region of the HDSS (Table A1).

Given that the participants in our analysis are between the ages of 15 and 64, we highlight changes in the population structure in 2011 (beginning of analysis follow-up time) and 2018 (end of analysis follow-up time). Overall, the were more women (54%) than men (46%). In 2018, compared to 2011, the population share of these individuals aged 15 to 64 years increased by one or two percentage points, except for those aged 20-24 and 25-29 years, whose shares declined by 4 and 3 percentage points, respectively. The overall mortality rate in the Gem region of Siaya HDSS decreased from 8.3 (95% CI: 7.5-9.2) per 1,000 person-years in 2011 to 5.1 (95% CI: 4.5-5.7) in 2018. All age groups between 15 and 64 saw a decline in age-specific mortality rates during this period; however, there was a threefold reduction in mortality rates for individuals aged 25 to 39 years (Table A2).

**Table A1. Mid-year population of the Siaya health and demographic surveillance system, from 2011 to 2018**

|  | **Siaya health and demographic surveillance system population** | **Gem region of the Siaya health and demographic surveillance system population** |
| --- | --- | --- |
| **Total mid-year population**  **(all ages)** |  |  |
| **Year** |  |  |
| 2011 | 260,701 | 94,689 |
| 2012 | 267,395 | 96,342 |
| 2013 | 277,065 | 98,497 |
| 2014 | 279,765 | 98,772 |
| 2015 | 277,950 | 98,306 |
| 2016 | 276,500 | 98,031 |
| 2017 | 274,452 | 98,159 |
| 2018 | 267,474 | 96,320 |

**Table A2 Age distribution and mortality rate (per 1000 person-years) of the population residing within Gem region of the Siaya health and demographic surveillance system**

|  | **Age distribution** | | **Mortality** | |
| --- | --- | --- | --- | --- |
| **Age** | **Number (%)** | **Number (%)** | **Mortality rate per 1,000 person-years**  **(95% CI)** | **Mortality rate per 1,000 person-years**  **(95% CI)** |
|  | **2011** | **2018** | **2011** | **2018** |
| 15-19 | 10,726 (23) | 12,394 (25) | 0.7 (0.4-1.5) | 1.2 (0.7-2.0) |
| 20-24 | 8,331 (18) | 6,978 (14) | 3.2 (2.2-4.7) | 2.1 (1.3-3.5) |
| 25-29 | 6,782 (14) | 5,414 (11) | 8.6 (6.6-11.1) | 3.7 (2.4-5.7) |
| 30-34 | 4,741 (10) | 5,537 (11) | 10.0 (7.5-13.2) | 3.6 (2.3-5.6) |
| 35-39 | 3,898 (8) | 4,755 (10) | 16.2 (12.6-20.7) | 6.9 (4.9-9.8) |
| 40-44 | 2,987 (6) | 3,857 (8) | 8.9 (6.1-13.0) | 8.1 (5.7-11.5) |
| 45-49 | 2,910 (6) | 3,107 (6) | 13.3 (9.7-18.2) | 7.7 (5.2-11.5) |
| 50-54 | 2,600 (5) | 2,776 (6) | 15.0 (11.0-20.4) | 9.3 (6.3-13.7) |
| 55-59 | 2,442 (5) | 2,788 (6) | 16.9 (12.5-23.0) | 9.0 (6.1-13.3) |
| 60-64 | 1,916 (4) | 2,264 (6) | 22.8 (17.0-30.5) | 19.5 (14.5-26.2) |
| **Overall** | **47,333** | **49,870** | **8.3 (7.5-9.2)** | **5.1 (4.5-5.7)** |

**Changes in the study population structure**

Of the 45,581 individuals that participated in the HIV serosurvey, 27,369 (60.0%) were part of first random sampling group, and 18,212 (40.0%) were in the second round of random sampling. The study population included all individuals aged 15 years and above who had at least one HIV test and resided in the Gem region of Siaya HDSS. During follow-up, 5,336 individuals reached age 65 and were excluded from the analysis; additionally, 1,386 died, and 10,863 migrated out of the area. The percentage of the population that participated in the first random sampling steadily declined over time compared to those that were included in the second round of sampling. The overall trend in out-migration and those that turned 65 years remained fairly stable over the years, whilst the number of those that turned 15 years, and death decreased over time (Table A3).

**HIV prevalence by random sampling groups in the HIV serosurveys**

There were slightly more females (59.1%) included in the first random sampling group, compared to those included in the second random sampling (53.9%) (Table A4). Differences in the percentage by age-groups tested for HIV were also observed. In the first random sample group, nearly half of the persons that were tested were aged 35 years and above, whilst in the second random sample group, only 31.2% of the people that were tested were in this age-group. Lastly, in the first random sample group, the percentage that were positive increased steadily across the years, whilst in the second random sample the percentage that were positive remained stable over time.

**Table A3. Annual changes in study population structure, Siaya health and demographic surveillance system**

|  | **End year study population** | | **Turned 15 years** | **Died** | **Out-migrated** | **Turned**  **65 years** |
| --- | --- | --- | --- | --- | --- | --- |
|  | **First random sampling** | **Second random sampling** |  |  |  |  |
|  | **Number (%)** | **Number (%)** | **Number** | **Number** | **Number** | **Number** |
| 2011 | 28,763 (100%) | 0 | 2,403 | 146 | 721 | 524 |
| 2012 | 24,051 (84.0%) | 4,567 (16.0%) | 2,695 | 204 | 1,156 | 464 |
| 2013 | 23,692 (74.2%) | 8,251 (25.8%) | 2,952 | 194 | 1,540 | 619 |
| 2014 | 22,388 (72.2%) | 8,640 (27.8%) | 2,550 | 192 | 1,698 | 768 |
| 2015 | 21,067 (71.6%) | 8,358 (28.4%) | 1,729 | 173 | 1,428 | 656 |
| 2016 | 20,120 (63.4%) | 12,023 (37.4%) | 1,649 | 179 | 1,335 | 458 |
| 2017 | 16,256 (57.5%) | 12,545 (43.6%) | 1,511 | 178 | 1,319 | 661 |
| 2018 | 14,347 (51.5%) | 14,054 (49.5%) | 1,764 | 120 | 1,666 | 843 |
| **Total** |  |  |  | **1,386** | **10,863** |  |

**Table A4. Distribution of sex, age, and HIV status by random sample groups in the HIV serosurvey, Siaya health and demographic surveillance system**

|  | **First random sampling (%)** | **Second random sampling (%)** |
| --- | --- | --- |
| **Sex** |  |  |
| Female | 59.1 | 53.9 |
| Male | 40.9 | 46.1 |
|  |  |  |
| **Age group** |  |  |
| 15-34 | 51.6 | 68.8 |
| 35-49 | 25.5 | 19.2 |
| 50-64 | 22.9 | 12.0 |
|  |  |  |
| **HIV status** |  |  |
| **2011** |  |  |
| Negative | 86.2 |  |
| Positive | 13.8 |  |
| **2012** |  |  |
| Negative | 86.4 | 86.4 |
| Positive | 13.6 | 13.6 |
| **2013** |  |  |
| Negative | 86.0 | 86.3 |
| Positive | 14.0 | 13.7 |
| **2014** |  |  |
| Negative | 85.7 | 87.1 |
| Positive | 14.3 | 12.9 |
| **2015** |  |  |
| Negative | 86.0 | 87.7 |
| Positive | 14.1 | 12.3 |
| **2016** |  |  |
| Negative | 83.7 | 86.2 |
| Positive | 16.3 | 13.8 |
| **2017** |  |  |
| Negative | 83.1 | 87.9 |
| Positive | 17.0 | 12.1 |
| **2018** |  |  |
| Negative | 80.2 | 86.7 |
| Positive | 19.8 | 13.3 |

**Age-group by cause of death over time**

HIV/AIDS/tuberculosis, infectious diseases and NCDs deaths were recorded across all age groups over time (Table A5). Meanwhile, external/obstetrics causes, and unclassifiable deaths were not observed across all age groups during some of the earlier years (2011-2014) of this analysis.

**Table A5. Number of people who have died, by cause of death, year, and age-group in the study population of the Siaya health and demographic surveillance system**

|  |  | 2011 | 2012 | 2013 | 2014 | 2015 | 2016 | 2017 | Total |
| --- | --- | --- | --- | --- | --- | --- | --- | --- | --- |
| **Age group** |  |  |  |  |  |  |  |  |  |
| **15-34 years** | HIV/AIDS/tuberculosis | 14 | 22 | 24 | 20 | 9 | 17 | 11 | 117 |
|  | Infectious diseases | 7 | 6 | 9 | 18 | 5 | 4 | 3 | 52 |
|  | Non-communicable diseases | 4 | 14 | 11 | 12 | 10 | 15 | 7 | 73 |
|  | External/obstetrics causes | 7 | 13 | 4 | 9 | 10 | 13 | 8 | 64 |
|  | Unclassifiable | 1 | **0** | 1 | **0** | 2 | 1 | 1 | 6 |
|  |  |  |  |  |  |  |  |  |  |
|  |  |  |  |  |  |  |  |  |  |
| **35-49 years** | HIV/AIDS/tuberculosis | 25 | 43 | 24 | 26 | 28 | 19 | 18 | 183 |
|  | Infectious diseases | 3 | 7 | 8 | 11 | 9 | 10 | 3 | 51 |
|  | Non-communicable diseases | 7 | 11 | 14 | 15 | 9 | 10 | 10 | 76 |
|  | External/obstetrics causes | **0** | 3 | 7 | 4 | 8 | 6 | 5 | 33 |
|  | Unclassifiable | 1 | **0** | **0** | 1 | 4 | 1 | 2 | 9 |
|  |  |  |  |  |  |  |  |  |  |
| **50-64 years** | HIV/AIDS/tuberculosis | 24 | 24 | 24 | 32 | 19 | 15 | 18 | 156 |
|  | Infectious diseases | 4 | 13 | 16 | 8 | 10 | 12 | 5 | 68 |
|  | Non-communicable diseases | 22 | 29 | 30 | 22 | 27 | 28 | 38 | 196 |
|  | External/obstetrics causes | 1 | **0** | 6 | 1 | 8 | 2 | 7 | 25 |
|  | Unclassifiable | **0** | 1 | 4 | 2 | 3 | 1 | 2 | 13 |
|  |  |  |  |  |  |  |  |  |  |
|  | **Total** | **120** | **186** | **182** | **181** | **161** | **154** | **138** | **1,122** |

*During the Joinpoint regression analysis, 0.5 was added to the figures in bold in Table A5; see further details in the Methods section under Joinpoint regression.

**Assessment of the number of joinpoints using Bayesian Information Criterion (BIC)**

Weighted BIC with improved computational efficiency was used to determine the number of joinpoints that were best suited to assess changes in mortality trends [1] (Table A6). The all-cause mortality model had eight data points (2011-2018), whilst the cause-specific mortality models had seven data points (2011-2017). The limited number of data points restricted the maximum number of joinpoints to one [2]. Based on the lowest BIC, no joinpoints were included except in the model of deaths due to infectious diseases.

**Table A6. Assessment of the number of joinpoints using Weighted Bayesian Information Criterion for the Joinpoint regression model**

|  | **Number of joinpoints** | **Persons with HIV** | **Persons without HIV** |
| --- | --- | --- | --- |
|  |  | Bayesian Information Criterion values | Bayesian Information Criterion values |
| All-cause | 0 | **1.04** | **1.14** |
|  | 1 | 1.13 | 1.35 |
|  |  |  |  |
| HIV/AIDS/tuberculosis | 0 | **0.53** | **0.96** |
|  | 1 | 0.55 | 1.32 |
|  |  |  |  |
| Infectious diseases | 0 | 1.10 | 0.82 |
|  | 1 | **0.73** | **-0.63** |
|  |  |  |  |
| Non-communicable diseases | 0 | **-0.83** | **0.28** |
|  | 1 | -0.39 | 0.51 |
|  |  |  |  |
| External/obstetrics causes | 0 | **0.31** | **-0.21** |
|  | 1 | 0.77 | -0.01 |

Figures in bold represent the lowest BIC values for each selected category.

**Assessment of the proportional hazard assumption in the Cox and competing risk regression models**

Among PWH, proportionality of mortality hazards across the years were violated by the age-group variable in the adjusted and unadjusted model for all-cause mortality (Table A6). In the adjusted model, the subdistribution hazard assumptions were violated for those with that had an unclassifiable death (age and sex variable) and those with a missing cause of death (year variable).

Among PWOH proportional subdistribution hazard assumption were only violated for those that had a missing cause of death in the multivariable model. In the univariable model, age-group violated the proportional hazard assumption in all-cause mortality, and calendar year violated among those were missing a cause of death. To relax the violation, an interaction with analysis time was included in this analysis.

**Table A7. Assessment of proportional hazard assumption using Schoenfeld residuals in the Cox and competing risk regression models**

|  | **People with HIV** | **People with HIV** | **People without HIV** | **People without HIV** |
| --- | --- | --- | --- | --- |
|  | Unadjusted model | Adjusted model | Unadjusted model | Adjusted model |
| **Cox Proportional Hazards Model** |  |  |  |  |
| **All-cause** |  |  |  |  |
| **Age-group** |  |  |  |  |
| 15-34 | Reference | Reference | Reference | Reference |
| 35-49 | **0.003** | **0.038** | 0.171 | 0.616 |
| *Time-varying hazard ratio* | *0.92 (0.87-0.97)* | *0.95 (0.89-1.00)* |  |  |
| 50-64 | **0.018** | 0.250 | **0.002** | 0.102 |
| *Time-varying hazard ratio* | *0.93 (0.88-0.99)* |  | *0.92 (0.87-0.97)* |  |
| **Sex** |  |  |  |  |
| Men | Reference | Reference | Reference | Reference |
| Women | 0.163 | 0.578 | 0.339 | 0.213 |
| **Year** | 0.886 | 0.924 | 0.755 | 0.951 |
|  |  |  |  |  |
| **Competing Risks Model** |  |  |  |  |
| **HIV/AIDS/tuberculosis** |  |  |  |  |
| **Age-group** |  |  |  |  |
| 15-34 | Reference | Reference | Reference | Reference |
| 35-49 | 0.131 | 0.409 | 0.855 | 0.968 |
| 50-64 | 0.321 | 0.892 | 0.286 | 0.404 |
| **Sex** |  |  |  |  |
| Men | Reference | Reference | Reference | Reference |
| Women | 0.151 | 0.537 | 0.959 | 0.169 |
| **Year** | 0.140 | 0.130 | 0.430 | 0.194 |
|  |  |  |  |  |
| **Infectious diseases** |  |  |  |  |
| **Age-group** |  |  |  |  |
| 15-34 | Reference | Reference | Reference | Reference |
| 35-49 | 0.335 | 0.641 | 0.448 | 0.341 |
| 50-64 | 0.178 | 0.519 | 0.902 | 0.831 |
| **Sex** |  |  |  |  |
| Men | Reference | Reference | Reference | Reference |
| Women | 0.085 | 0.143 | 0.203 | 0.687 |
| **Year** | 0.965 | 0.896 | 0.841 | 0.764 |
|  |  |  |  |  |
| **Non-communicable diseases** |  |  |  |  |
| **Age-group** |  |  |  |  |
| 15-34 | Reference | Reference | Reference | Reference |
| 35-49 | **0.055** | 0.126 | 0.632 | 0.856 |
| *Time-varying hazard ratio* | *0.85 (0.71-1.00)* |  |  |  |
| 50-64 | 0.659 | 0.886 | 0.142 | 0.376 |
| **Sex** |  |  |  |  |
| Men | Reference | Reference | Reference | Reference |
| Women | 0.179 | 0.631 | 0.702 | 0.177 |
| **Year** | 0.309 | 0.271 | 0.386 | 0.810 |
|  |  |  |  |  |
| **External/obstetric causes** |  |  |  |  |
| **Age-group** |  |  |  |  |
| 15-34 | Reference | Reference | Reference | Reference |
| 35-49 | 0.134 | 0.070 | 0.260 | 0.498 |
| 50-64 | 0.273 | 0.567 | 0.392 | 0.161 |
| **Sex** |  |  |  |  |
| Men | Reference | Reference | Reference | Reference |
| Women | 0.440 | 0.307 | 0.256 | 0.139 |
| **Year** | 0.825 | 0.940 | 0.912 | 0.658 |
|  |  |  |  |  |
| **Unclassifiable** |  |  |  |  |
| **Age-group** |  |  |  |  |
| 15-34 | Reference | Reference | Reference | Reference |
| 35-49 | 0.101 | 0.084 | 0.971 | 0.991 |
| 50-64 | 0.078 | **0.034** | 0.698 | 0.796 |
| *Time-varying hazard ratio* |  | *0.77 (0.61-0.98)* |  |  |
| **Sex** |  |  |  |  |
| Men | Reference | Reference | Reference | Reference |
| Women | **0.018** | **0.003** | 0.114 | 0.269 |
| *Time-varying hazard ratio* | *0.70 (0.53-0.94)* | *0.65 (0.48-0.86)* |  |  |
| **Year** | 0.445 | 0.297 | 0.860 | 0.973 |
|  |  |  |  |  |
| **Missing** |  |  |  |  |
| **Age-group** |  |  |  |  |
| 15-34 | Reference | Reference | Reference | Reference |
| 35-49 | 0.455 | 0.533 | 0.469 | 0.698 |
| 50-64 | 0.466 | 0.647 | 0.088 | 0.289 |
| **Sex** |  |  |  |  |
| Men | Reference | Reference | Reference | Reference |
| Women | 0.541 | 0.722 | 0.430 | 0.960 |
| **Year** | **<0.001** | **<0.001** | 0.058 | **0.043** |
| *Time-varying hazard ratio* | *1.07 (1.03-1.10)* | *1.07 (1.03-1.10)* |  | *1.08 (1.00-1.17)* |
|  |  |  |  |  |

P values in bold are accompanied by hazard ratio and 95% CI where the proportional sub-distribution hazards assumption was violated

**Table A8. Grouped causes of death in the study population of the Siaya health demographic surveillance system from 2011 to 2018**

| **Broad category** | **Detailed conditions** | **Dead (%)** |
| --- | --- | --- |
| **HIV/AIDS related death** | HIV/AIDS related death | 312 (27.8) |
|  | Pulmonary tuberculosis | 144 (12.8) |
| **Infectious diseases** | Acute respiratory infection including pneumonia | 98 (8.7) |
|  | Malaria | 41 (3.7) |
|  | Meningitis and encephalitis | 21 (1.9) |
|  | Other and unspecified infectious diseases | 5 (0.4) |
|  | Diarrhoeal diseases | 4 (0.4) |
|  | Sepsis (non-obstetric) | 2 (0.2) |
| **Non-communicable diseases** | Acute abdomen | 53 (4.7) |
|  | Digestive neoplasms | 38 (3.4) |
|  | Respiratory neoplasms | 37 (3.3) |
|  | Stroke | 29 (2.6) |
|  | Acute cardiac disease | 25 (2.2) |
|  | Epilepsy | 25 (2.2) |
|  | Other and unspecified cardiac disease | 23 (2.0) |
|  | Other and unspecified neoplasms | 20 (1.8) |
|  | Chronic obstructive pulmonary disease | 20 (1.8) |
|  | Asthma | 17 (1.5) |
|  | Reproductive neoplasms | 16 (1.4) |
|  | Diabetes mellitus | 15 (1.3) |
|  | Liver cirrhosis | 6 (0.5) |
|  | Renal failure | 5 (0.4) |
|  | Severe anaemia | 4 (0.4) |
|  | Severe malnutrition | 4 (0.4) |
|  | Breast neoplasms | 3 (0.3) |
|  | Other and unspecified non-communicable diseases | 3 (0.3) |
|  | Oral neoplasms | 2 (0.2) |
| **Direct obstetrics-related** | Obstetric haemorrhage | 7 (0.6) |
|  | Pregnancy-induced hypertension | 2 (0.2) |
|  | Abortion-related death | 1 (0.1) |
|  | Obstructed labour | 1 (0.1) |
|  | Pregnancy-related sepsis | 1 (0.1) |
|  | Anaemia of pregnancy | 1 (0.1) |
|  | Ectopic pregnancy | 1 (0.1) |
| **External causes of death** | Assault | 49 (4.4) |
|  | Road traffic accident | 28 (2.5) |
|  | Accidental drowning and submersion | 8 (0.7) |
|  | Accidental fall | 7 (0.6) |
|  | Intentional self-harm | 7 (0.6) |
|  | Other and unspecified external causes | 4 (0.4) |
|  | Accidental exposure to smoke fire & flame | 2 (0.2) |
|  | Contact with venomous plant/animal | 1 (0.1) |
|  | Accidental poisoning & noxious substance | 1 (0.1) |
|  | Exposure to force of nature | 1 (0.1) |
| **Unclassifiable** | Indeterminate | 28 (2.5) |
| **Total** |  | **1,122** |

**All-cause and cause-specific mortality patterns in the Cox and competing risks models**

In the unadjusted and adjusted model for sex and age, all-cause and cause-specific mortality hazards increased with advancing age among PWH and PWOH (Table A9). By sex, the mortality hazards tended to be lower in women compared to men for both PWH and PWOH.

**Table A9.** **All-cause and cause-specific mortality hazard ratios (95% confidence interval) for age, sex, and calendar year in the study population of the Siaya health and demographic surveillance system from 2011-2018**

|  | **People with HIV** | **People with HIV** | **People without HIV** | **People without HIV** |
| --- | --- | --- | --- | --- |
|  | **Unadjusted** | **Adjusted** | **Unadjusted** | **Adjusted** |
| **All-cause** |  |  |  |  |
| **Age** |  |  |  |  |
|  |  |  |  |  |
| 15-34 | 1.00 | 1.00 | 1.00 | 1.00 |
| 35-49 | 1.22 (1.02-1.48) | 1.14 (0.94-1.37) | 1.99 (1.60-2.46) | 2.20 (1.78-2.73) |
| 50-64 | 1.55 (1.27-1.91) | 1.42 (1.16-1.74) | 5.27 (4.43-6.26) | 5.93 (4.99-7.06) |
| **Sex** |  |  |  |  |
| Men | 1.00 | 1.00 | 1.00 | 1.00 |
| Women | 0.47 (0.40-0.55) | 0.49 (0.42-0.57) | 0.65 (0.56-0.75) | 0.50 (0.43-0.58) |
| **Year** | 0.86 (0.81-0.92) | 0.88 (0.83-0.93) | 0.92 (0.86-0.98) | 0.96 (0.90-1.02) |
| **HIIV/AIDS/tuberculosis** |  |  |  |  |
| **Age** |  |  |  |  |
| 15-34 | 1.00 | 1.00 | 1.00 | 1.00 |
| 35-49 | 1.45 (1.10-1.90) | 1.34 (1.02-1.76) | 4.14 (2.50-6.84) | 4.39 (2.65-7.27) |
| 50-64 | 1.55 (1.13-2.11) | 1.38 (1.02-1.88) | 8.76 (5.57-13.78) | 9.12 (5.75-14.45) |
| **Sex** |  |  |  |  |
| Men | 1.00 | 1.00 | 1.00 | 1.00 |
| Women | 0.48 (0.38-0.60) | 0.51 (0.41-0.64) | 0.89 (0.64-1.25) | 0.66 (0.47-0.93) |
| **Year** | 0.81 (0.76-0.87) | 0.83 (0.78-0.89) | 0.80 (0.70-0.91) | 0.85 (0.75-0.96) |
| **Infectious diseases** |  |  |  |  |
| **Age** |  |  |  |  |
| 15-34 | 1.00 | 1.00 | 1.00 | 1.00 |
| 35-49 | 0.98 (0.57-1.71) | 0.94 (0.54-1.61) | 2.27 (1.28-4.01) | 2.42 (1.35-4.33) |
| 50-64 | 1.39 (0.76-2.53) | 1.31 (0.73-2.36) | 5.15 (3.16-8.39) | 5.32 (3.19-8.89) |
| **Sex** |  |  |  |  |
| Men | 1.00 | 1.00 | 1.00 | 1.00 |
| Women | 0.55 (0.35-0.86) | 0.56 (0.36-0.87) | 0.79 (0.52-1.19) | 0.63 (0.41-0.96) |
| **Year** | 0.95 (0.85-1.06) | 0.97 (0.87-1.08) | 0.73 (0.64-0.83) | 0.77 (0.68-0.87) |
| **Non-communicable diseases** |  |  |  |  |
| **Age** |  |  |  |  |
| 15-34 | 1.00 | 1.00 | 1.00 | 1.00 |
| 35-49 | 1.78 (1.01-3.13) | 1.62 (0.93-2.82) | 1.91 (1.24-2.92) | 2.12 (1.38-3.24) |
| 50-64 | 3.99 (2.30-6.92) | 3.56 (2.07-6.13) | 8.57 (6.22-11.79) | 9.64 (7.03-13.23) |
| **Sex** |  |  |  |  |
| Men | 1.00 | 1.00 | 1.00 | 1.00 |
| Women | 0.42 (0.29-0.62) | 0.46 (0.32-0.68) | 0.65 (0.50-0.84) | 0.48 (0.37-0.63) |
| **Year** | 0.93 (0.84-1.03) | 0.96 (0.87-1.06) | 0.93 (0.84-1.04) | 0.99 (0.89-1.09) |
| **External/obstetrics causes** |  |  |  |  |
| **Age** |  |  |  |  |
| 15-34 | 1.00 | 1.00 | 1.00 | 1.00 |
| 35-49 | 0.56 (0.26-1.19) | 0.52 (0.24-1.10) | 1.26 (0.76-2.11) | 1.53 (0.92-2.52) |
| 50-64 | 0.27 (0.08-1.00) | 0.26 (0.07-0.93) | 1.31 (0.79-2.18) | 1.64 (1.00-2.67) |
| **Sex** |  |  |  |  |
| Men | 1.00 | 1.00 | 1.00 | 1.00 |
| Women | 0.40 (0.19-0.85) | 0.37 (0.17-0.79) | 0.31 (0.20-0.49) | 0.29 (0.19-0.45) |
| **Year** | 1.01 (0.83-1.24) | 1.03 (0.84-1.26) | 0.94 (0.83-1.07) | 0.97 (0.85-1.09) |
| **Unclassifiable** |  |  |  |  |
| **Age** |  |  |  |  |
| 15-34 | 1.00 | 1.00 | 1.00 | 1.00 |
| 35-49 | 2.11 (0.31-14.34) | 1.81 (0.28-11.67) | 3.01 (0.77-11.85) | 3.16 (0.82-12.16) |
| 50-64 | 3.79 (0.57-25.18) | 3.23 (0.51-20.39) | 6.55 (1.94-22.14) | 6.88 (2.06-22.96) |
| **Sex** |  |  |  |  |
| Men | 1.00 | 1.00 | 1.00 | 1.00 |
| Women | 0.24 (0.08-0.76) | 0.26 (0.08-0.87) | 0.78 (0.28-2.16) | 0.64 (0.25-1.66) |
| **Year** | 1.02 (0.80-1.31) | 1.07 (0.85-1.35) | 0.94 (0.73-1.23) | 0.98 (0.77-1.26) |
| **Missing** |  |  |  |  |
| **Age** |  |  |  |  |
| 15-34 | 1.00 | 1.00 | 1.00 | 1.00 |
| 35-49 | 0.71 (0.42-1.23) | 0.70 (0.41-1.20) | 1.32 (0.64-2.69) | 1.48 (0.73-3.01) |
| 50-64 | 0.94 (0.52-1.70) | 0.91 (0.51-1.64) | 3.67 (2.10-6.43) | 4.11 (2.40-7.06) |
| **Sex** |  |  |  |  |
| Men | 1.00 | 1.00 | 1.00 | 1.00 |
| Women | 0.78 (0.49-1.22) | 0.78 (0.49-1.24) | 0.48 (0.29-0.80) | 0.41 (0.25-0.68) |
| **Year** | 0.90 (0.82-1.00) | 0.91 (0.82-1.01) | 0.82 (0.63-1.06) | 0.87 (0.68-1.10) |

**Table A10. All-cause and cause-specific mortality hazard ratios (95% confidence interval) for age, sex, calendar year, and interaction of HIV status and calendar year in the study population of the Siaya health and demographic surveillance system from 2011-2018**

|  | **Adjusted hazard ratios** |
| --- | --- |
| **All-cause** |  |
| **HIV status** |  |
| Negative | 1.00 |
| Positive | 4.86 (4.34-5.45) |
| **Age** |  |
| 15-34 | 1.00 |
| 35-49 | 1.77 (1.54-2.04) |
| 50-64 | 3.34 (2.93-3.82) |
| **Sex** |  |
| Men | 1.00 |
| Women | 0.53 (0.47-0.59) |
| Year | 0.96 (0.91-1.01) |
| **Interaction Term (HIV#Year)** | 0.91 (0.87-0.96) |
| **HIIV/AIDS/tuberculosis** |  |
| **HIV status** |  |
| Negative | 1.00 |
| Positive | 11.26 (8.72-14.54) |
| **Age** |  |
| 15-34 | 1.00 |
| 35-49 | 2.04 (1.58-2.63) |
| 50-64 | 2.78 (2.15-3.61) |
| **Sex** |  |
| Men | 1.00 |
| Women | 0.60 (0.50-0.72) |
| Year | 0.87 (0.79-0.95) |
| **Interaction Term (HIV#Year)** | 0.94 (0.85-1.04) |
| **Infectious diseases** |  |
| **HIV status** |  |
| Negative | 1.00 |
| Positive | 4.46 (3.09-6.44) |
| **Age** |  |
| 15-34 | 1.00 |
| 35-49 | 1.66 (1.09-2.53) |
| 50-64 | 3.12 (2.09-4.64) |
| **Sex** |  |
| Men | 1.00 |
| Women | 0.64 (0.47-0.87) |
| Year | 0.88 (0.78-0.99) |
| **Interaction Term (HIV#Year)** | 1.01 (0.88-1.17) |
| **Non-communicable diseases** |  |
| **HIV status** |  |
| Negative | 1.00 |
| Positive | 2.25 (1.76-2.89) |
| **Age** |  |
| 15-34 | 1.00 |
| 35-49 | 2.21 (1.59-3.07) |
| 50-64 | 7.57 (5.73-10.00) |
| **Sex** |  |
| Men | 1.00 |
| Women | 0.49 (0.40-0.61) |
| Year | 0.98 (0.90-1.06) |
| **Interaction Term (HIV#Year)** | 0.99 (0.89-1.10) |
| **External/obstetrics causes** |  |
| **HIV status** |  |
| Negative | 1.00 |
| Positive | 1.84 (1.14-2.98) |
| **Age** |  |
| 15-34 | 1.00 |
| 35-49 | 1.17 (0.73-1.85) |
| 50-64 | 1.13 (0.70-1.84) |
| **Sex** |  |
| Men | 1.00 |
| Women | 0.33 (0.23-0.48) |
| Year | 1.01 (0.90-1.13) |
| **Interaction Term (HIV#Year)** | 0.95 (0.78-1.16) |
| **Unclassifiable** |  |
| **HIV status** |  |
| Negative | 1.00 |
| Positive | 3.52 (1.57-7.91) |
| **Age** |  |
| 15-34 | 1.00 |
| 35-49 | 2.85 (0.95-8.55) |
| 50-64 | 5.79 (2.10-16.00) |
| **Sex** |  |
| Men | 1.00 |
| Women | 0.45 (0.21-0.95) |
| Year | 0.96 (0.75-1.23) |
| **Interaction Term (HIV#Year)** | 1.08 (0.76-1.55) |
| **Missing** |  |
| **HIV status** |  |
| Negative | 1.00 |
| Positive | 7.20 (4.94-10.48) |
| **Age** |  |
| 15-34 | 1.00 |
| 35-49 | 1.01 (0.65-1.59) |
| 50-64 | 1.92 (1.25-2.94) |
| **Sex** |  |
| Men | 1.00 |
| Women | 0.62 (0.45-0.86) |
| Year | 0.84 (0.72-0.97) |
| **Interaction Term (HIV#Year)** | 1.09 (0.94-1.28) |

**Table A11. Varying assumptions about person-time before the last negative HIV test (2 years or 5 years) among persons without HIV in the study population of the Siaya health and demographic surveillance system from 2011-2018**

|  | **Causes of death** | | | | | | |
| --- | --- | --- | --- | --- | --- | --- | --- |
|  | **All-cause** | **HIV/AIDS/**  **tuberculosis** | **Infectious**  **diseases** | **Non-communicable**  **diseases** | **External/obstetrics causes** | **Unclassifiable** | **Missing** |
|  | **Hazard ratio**  **(95% CI)** | **Subdistribution**  **hazard ratio**  **(95% CI)** | **Subdistribution**  **hazard ratio**  **(95% CI)** | **Subdistribution**  **hazard ratio**  **(95% CI)** | **Subdistribution**  **hazard ratio**  **(95% CI)** | **Subdistribution**  **hazard ratio (95% CI)** | **Subdistribution**  **hazard ratio (95% CI)** |
| **Persons without HIV**  **Unadjusted** |  |  |  |  |  |  |  |
| **Time to seroconversion: 2 years** | 0.90  (0.83-0.97) | 0.74  (0.64-0.85) | 0.72  (0.62-0.85) | 0.93  (0.82-1.05) | 0.84  (0.69-1.01) | 0.80  (0.57-1.13) | 0.72  (0.52-1.01) |
| **Time to seroconversion: 5 years** | 0.92  (0.86-0.98) | 0.80  (0.70-0.91) | 0.73  (0.64-0.83) | 0.93  (0.84-1.04) | 0.94  (0.83-1.07) | 0.94  (0.73-1.23) | 0.82  (0.63-1.06) |

**Reference**

1. Liu B, Kim H-J, Feuer EJ, Graubard BI. Joinpoint Regression Methods of Aggregate Outcomes for Complex Survey Data. Journal of Survey Statistics and Methodology. 2022;11(4):967-89.

2. Joinpoint Regression Program. Number of Joinpoints USA: National Cancer Institute. Division of Cancer Control and Population Sciences; 2024 [cited 2024 13 Decemeber 2024]. Available from: <https://surveillance.cancer.gov/help/joinpoint/setting-parameters/method-and-parameters-tab/number-of-joinpoints>.
